# Supplementation with Exogenous Catalase from *Penicillium notatum* in the Diet Ameliorates Lipopolysaccharide-Induced Intestinal Oxidative Damage through Affecting Intestinal Antioxidant Capacity and Microbiota in Weaned Pigs

**DOI:** 10.1128/Spectrum.00654-21

**Published:** 2021-12-15

**Authors:** Jiali Chen, Fuchang Li, Weiren Yang, Shuzhen Jiang, Yang Li

**Affiliations:** a Shandong Provincial Key Laboratory of Animal Biotechnology and Disease Control and Prevention, Department of Animal Science and Veterinary Medicine, Shandong Agricultural University, Tai’an, China; USDA-ARS, ACNC

**Keywords:** weaned pigs, catalase, intestine, oxidative stress, microorganisms, lipopolysaccharide

## Abstract

The present study aimed to explore the protective effects of exogenous catalase (CAT) from microorganisms against lipopolysaccharide (LPS)-induced intestinal injury and its molecular mechanism in weaned pigs. Fifty-four weaned pigs (21 days of age) were randomly allocated to CON, LPS, and LPS+CAT groups. The pigs in CON and LPS groups were fed a basal diet, whereas the pigs in LPS+CAT group fed the basal diet with 2,000 mg/kg CAT supplementation for 35 days. On day 36, six pigs were selected from each group, and LPS and LPS+CAT groups were administered with LPS (50 μg/kg body weight). Meanwhile, CON group was injected with an equivalent amount of sterile saline. Results showed that LPS administration damaged intestinal mucosa morphology and barrier. However, CAT supplementation alleviated the deleterious effects caused by LPS challenge through enhancing intestinal antioxidant capacity which was benefited to decrease proinflammatory cytokines concentrations and suppress enterocyte apoptosis. Besides, LPS-induced gut microbiota dysbiosis was significantly shifted by CAT through decreasing mainly Streptococcus and Escherichia-Shigella. Our study suggested that dietary supplemented with 2,000 mg/kg catalase was conducive to improve intestinal development and protect against LPS-induced intestinal mucosa injury via enhancing intestinal antioxidant capacity and altering microbiota composition in weaned pigs.

**IMPORTANCE** Exogenous CAT derived from microorganisms has been widely used in food, medicine, and other industries. Recent study also found that exogenous CAT supplementation could improve growth performance and antioxidant capacity of weaned pigs. However, it is still unknown that whether dietary exogenous CAT supplementation can provide a defense against the oxidative stress-induced intestinal damage in weaned pigs. Our current study suggested that dietary supplemented with 2,000 mg/kg CAT was conducive to improve intestinal development and protect against LPS-induced intestinal mucosa injury via enhancing intestinal antioxidant capacity and altering microbiota composition in weaned pigs. Moreover, this study will also assist in developing of CAT produced by microorganisms to attenuate various oxidative stress-induced injury or diseases.

## INTRODUCTION

The intestine plays a vital role in the digestion and absorption of nutrients, and the intestinal epithelial barrier serves as the first line against harmful pathogens (1). Numerous scientific evidences showed that alteration of the intestinal barrier contributed to increased intestinal permeability that allowed the passage of endotoxins and other bacterial products to the blood, and development of numerous gastrointestinal/extra-intestinal diseases including inflammatory bowel disease, cardiovascular disease, and mineral metabolism disorders (2, 3). The weaning process is one of the most stressful periods in the pig’s life. During this time, weaned pigs are subjected to multiple stress factors such as weaning, environmental factors, and infection, which leads to overproduction of reactive oxygen species (ROS) resulting in destruction of the equilibrium between ROS generation and antioxidant capacity of the body (4, 5). As a consequence, oxidative stress occurs. The intestine is highly vulnerable to oxidative stress injury because the intestine cells contain a large number of mitochondria that are the major sites for ROS production (6, 7). Oxidative stress has been proven to cause cell apoptosis, impaired intestinal development, and intestinal flora disorder that result in reduced pig health and growth (8–10). Therefore, it is very important to alleviate the post-weaning oxidative stress for pigs.

Hydrogen peroxide (H_2_O_2_), one of the most abundant ROS, can induce detrimental oxidative damage to cellular structures, nucleic acids, proteins, and membrane lipids when concentrations are above the critical levels (11). Catalase (CAT) serves as an important enzyme in the body and can protect organisms against oxidative stress through eliminating H_2_O_2_ by breaking down H_2_O_2_ into oxygen and water (8). At present, exogenous CAT extracted from microorganisms is increasingly used in pig production to protect against oxidative stress (8, 12). Recent studies showed that dietary supplementation with exogenous CAT could improve growth performance, promote intestinal development, and relieve hepatic oxidative damage induced by lipopolysaccharide (LPS) challenge through enhancing antioxidant capacity in weaned pigs (8, 12, 13). However, very little published information is available regarding whether dietary exogenous CAT supplementation can alleviate intestinal damage under oxidative stress condition in weaned pigs. The LPS, a main component of the outer membranes of Gram-negative bacteria, is a potent endotoxin releasing ROS *in vivo* (14). LPS-challenged animals are excellent models for studying oxidative stress and intestinal injury (15, 16). Thus, our study aimed to explore whether supplementation of exogenous CAT in the diet could alleviate intestinal damage and to elucidate its underlying mechanism in LPS-challenged weaned pigs.

## RESULTS

### Intestinal morphology.

**FIG 1:**
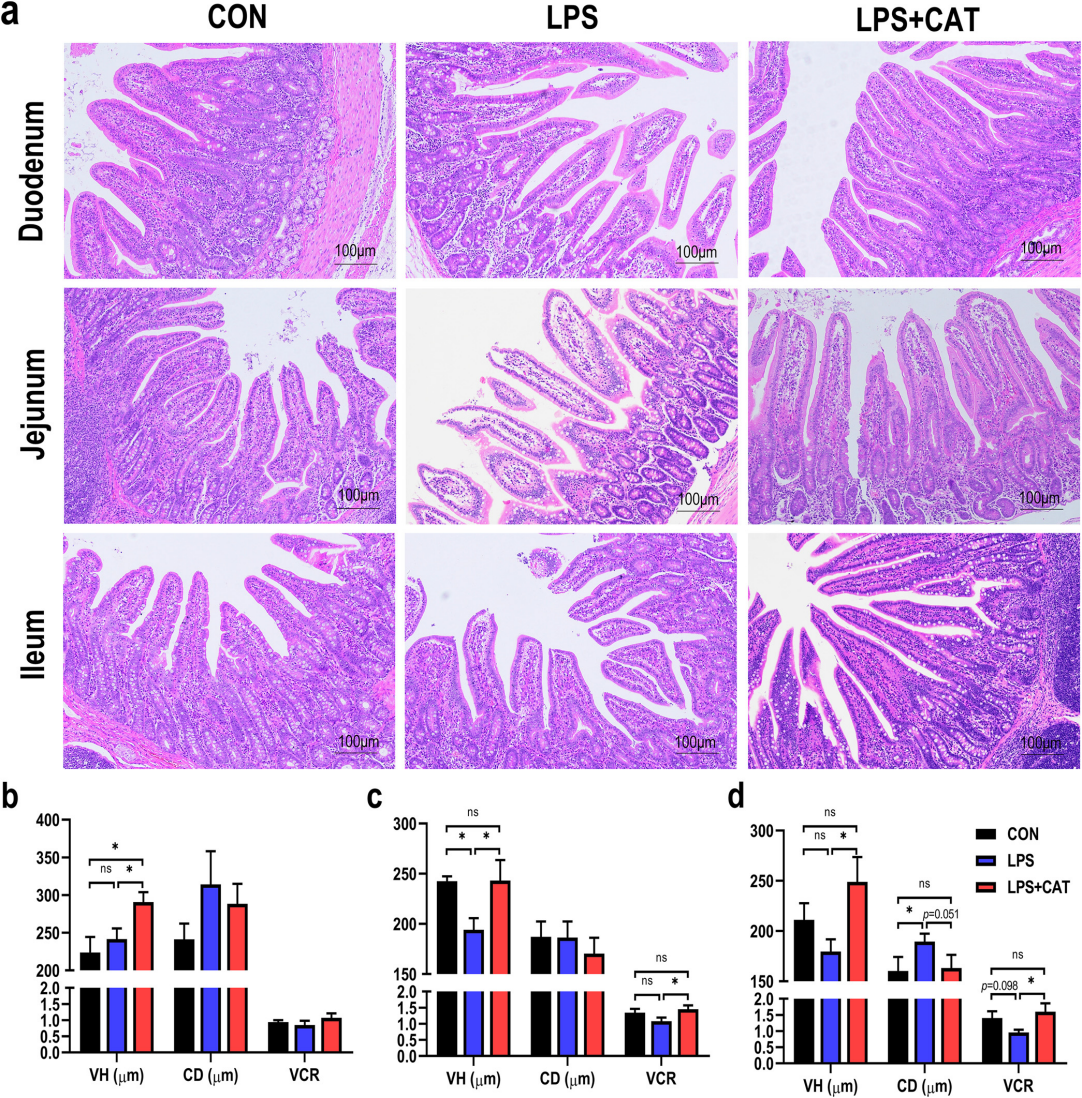
Effects of dietary catalase (CAT) supplementation on intestinal morphology in weaned pigs challenged with lipopolysaccharide (LPS). (a) Hematoxylin and eosin photomicrographs obtained at 100× magnification. (b) duodenum; (c) jejunum; (d) ileum. CON, pigs fed the basal diet and given intraperitoneal administration of saline solution; LPS, pigs fed the basal diet and given intraperitoneal administration of LPS; LPS+CAT, pigs fed the basal diet supplemented with 2,000 mg/kg exogenous catalase and given intraperitoneal administration of LPS. VH, villus height; CD, crypt depth; VCR, the ratio of villus height to crypt depth. The statistical analysis was performed using one-way analysis of variance, and the differences among group means were compared using the least significant difference method. Values are mean ± standard error (*N* = 6). Significant differences are displayed in the figures by * *P* < 0.05, while 0.05 < *P* < 0.10 was considered as a trend to significance. Nonsignificant differences are indicated by “ns.”

Intestinal mucosa injuries, such as villus shedding and atrophy, were found in the LPS-challenged pigs, but supplementation with dietary CAT seemed to attenuate LPS-induced intestinal mucosal damage (Fig. 1a). As shown in Fig. 1b, the duodenal villus height (VH) of pigs in the LPS+CAT group was markedly higher than those of pigs in the CON and LPS groups (*P < *0.05). In jejunum (Fig. 1c), relative to the pigs in CON group, the pigs in LPS group showed significantly decreased VH (*P* < 0.05). In LPS-challenged pigs, dietary CAT addition significantly increased the VH and the ratio of villus height to crypt depth (VCR) of pigs (*P < *0.05). In ileum (Fig. 1d), compared with the CON pigs, LPS pigs had a higher crypt depth (CD) (*P <* 0.05) and a lower VCR (*P = *0.098). Dietary CAT supplementation significantly enhanced the VH and VCR (*P* < 0.05) and tended to decrease the CD compared in pigs injected with LPS (*P = *0.051). There were no significant differences in jejunum and ileum indexes between CON pigs and LPS+CAT pigs (*P >* 0.05).

### Intestinal mucosal barrier maturity and integrity.

**FIG 2:**
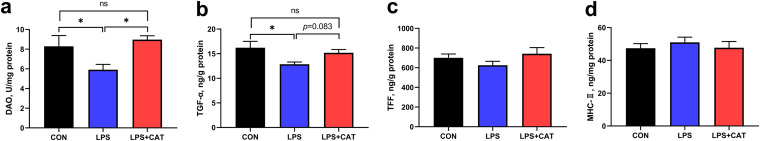
Effects of dietary catalase (CAT) supplementation on the maturity and integrity of jejunum mucosal barrier in weaned pigs challenged with lipopolysaccharide (LPS). (a) DAO, diamine oxidase; (b) TGF-α, transforming growth factor-α; (c) TFF, trefoil factor family; (d) MCH-II, major histocompatibility complex class II. CON, pigs fed the basal diet and given intraperitoneal administration of saline solution; LPS, pigs fed the basal diet and given intraperitoneal administration of LPS; LPS+CAT, pigs fed the basal diet supplemented with 2,000 mg/kg exogenous CAT and given intraperitoneal administration of LPS. The statistical analysis was performed using one-way analysis of variance, and the differences among group means were compared using the least significant difference method. Values are mean ± standard error (*N*  =  6). Significant differences are displayed in the figures by * *P  < * 0.05, while 0.05  <  *P  <  *0.10 was considered as a trend to significance. Nonsignificant differences are indicated by “ns.”

As shown in Fig. 2, significantly decreased jejunal mucosa diamine oxidase (DAO) and transforming growth factor-α (TGF-α) concentrations were observed in LPS pigs compared with the CON pigs (*P < *0.05). Dietary supplementation of 2,000 mg/kg CAT significantly increased the DAO concentration (*P < *0.05) and tended to elevate the TGF-α concentration (*P* < 0.083) in LPS-challenged pigs, and there were no significant differences in DAO and TGF-α concentrations between the CON group and the LPS+CAT group (*P > *0.05). Jejunal mucosa trefoil factor family (TFF) and major histocompatibility complex class II (MHC-II) concentrations showed no significant differences among the three treatments (*P > *0.05).

### Intestinal antioxidative capacity.

**FIG 3:**
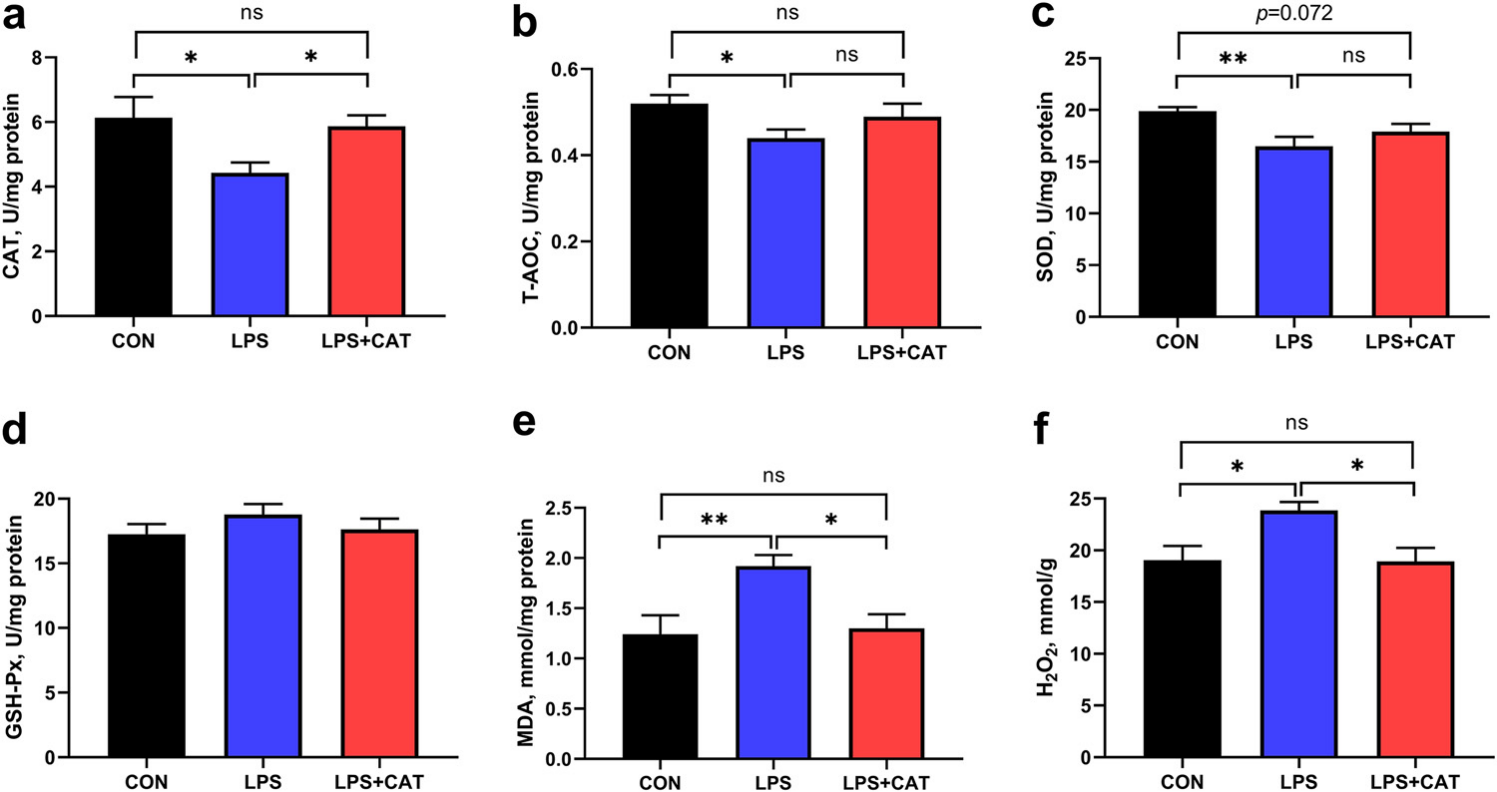
Effects of dietary catalase (CAT) supplementation on oxidant and antioxidant indicators in jejunal mucosa of weaned pigs challenged with lipopolysaccharide (LPS). (a) CAT; (b) T-AOC, total antioxidant capacity; (c) SOD, superoxide dismutase; (d) GSH-Px, glutathione peroxidase; (e) MDA, malondialdehyde; (f) H_2_O_2_, hydrogen peroxide. CON, pigs fed the basal diet and given intraperitoneal administration of saline solution; LPS, pigs fed the basal diet and given intraperitoneal administration of LPS; LPS+CAT, pigs fed the basal diet supplemented with 2,000 mg/kg exogenous CAT and given intraperitoneal administration of LPS. The statistical analysis was performed using one-way analysis of variance, and the differences among group means were compared using the least significant difference method. Values are mean ± standard error (*N* = 6). Significant differences are displayed in the figures by * *P* < 0.05 and ** *P < *0.01, while 0.05 < *P* < 0.10 was considered as a trend to significance. Nonsignificant differences are indicated by “ns.”

Compared with the CON pigs, LPS pigs exhibited significantly reduced total antioxidant capacity (T-AOC), CAT activity, and superoxide dismutase (SOD), activity (*P* < 0.05), and significantly increased malondialdehyde (MDA) and H_2_O_2_ concentrations (*P* < 0.05) (Fig. 3). In LPS-challenged pigs, dietary supplementation of exogenous CAT significantly enhanced the activity of CAT and the concentrations of MDA and H_2_O_2_ in jejunal mucosa (*P <* 0.05). Dietary supplementation of 2,000 mg/kg CAT also tended to ameliorate LPS-induced decrease in SOD activity (*P = *0.072). There were no significant differences in activities of CAT and glutathione peroxidase (GSH-Px), T-AOC, and concentrations of MDA and H_2_O_2_ between CON pigs and LPS+CAT pigs (*P >* 0.05).

### Intestinal inflammatory cytokines and secretory immunoglobulin A concentrations.

**FIG 4:**
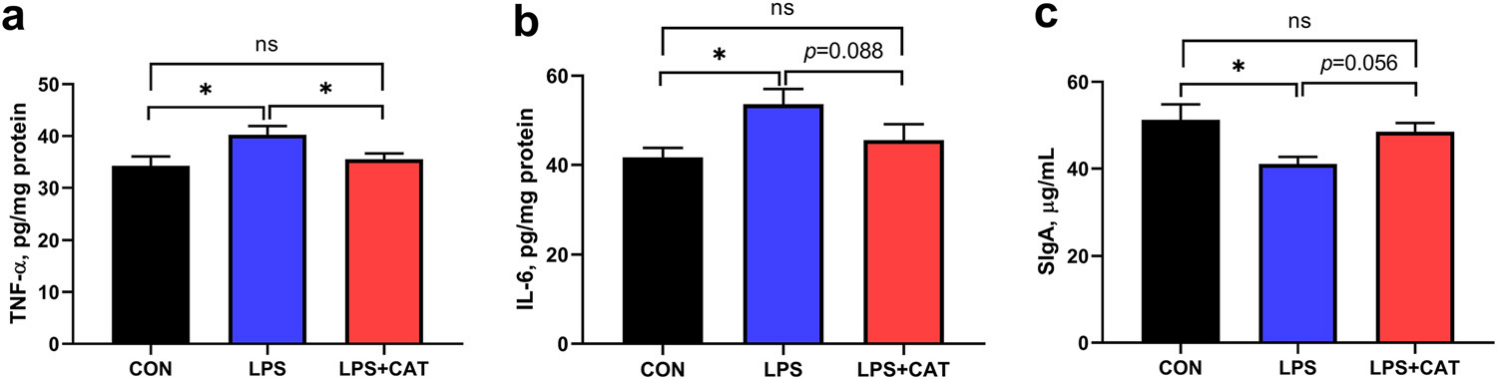
Effects of supplementation with dietary catalase (CAT) on the levels of inflammatory cytokines and SIgA in jejunal mucosa of weaned pigs challenged with lipopolysaccharide (LPS). (a) TNF-α, tumor necrosis factor-α; (b) IL-6, interleukin-6; (c) SIgA, secretory immunoglobulin A. CON, pigs fed the basal diet and given intraperitoneal administration of saline solution; LPS, pigs fed the basal diet and given intraperitoneal administration of LPS; LPS+CAT, pigs fed the basal diet supplemented with 2,000 mg/kg exogenous CAT and given intraperitoneal administration of LPS. The statistical analysis was performed using one-way analysis of variance, and the differences among group means were compared using the least significant difference method. Values are mean ± standard error (N = 6). Significant differences are displayed in the figures by * *P <* 0.05, while 0.05 < *P* < 0.10 was considered as a trend to significance. Non-significant differences are indicated by “ns.”

As shown in Fig. 4, compared with CON group, LPS administration resulted in significant increases in tumor necrosis factor-α (TNF-α) and interleukin-6 (IL-6) concentrations (*P <* 0.05) and a significant reduction in secretory immunoglobulin A (SIgA) concentration (*P <* 0.05) in jejunal mucosa. Dietary supplementation with 2,000 mg/kg CAT decreased TNF-α concentration by 11.82% (*P* < 0 .05) and IL-6 concentration by 15.0% (*P = *0.088), and increased SIgA concentration by 18.14% (*P =* 0.056) compared with LPS pigs. No significant differences were observed in concentrations of TNF-α, IL-6, and SIgA between the CON group and the LPS+CAT group (*P > *0.05).

### Intestinal caspase activity.

**FIG 5:**
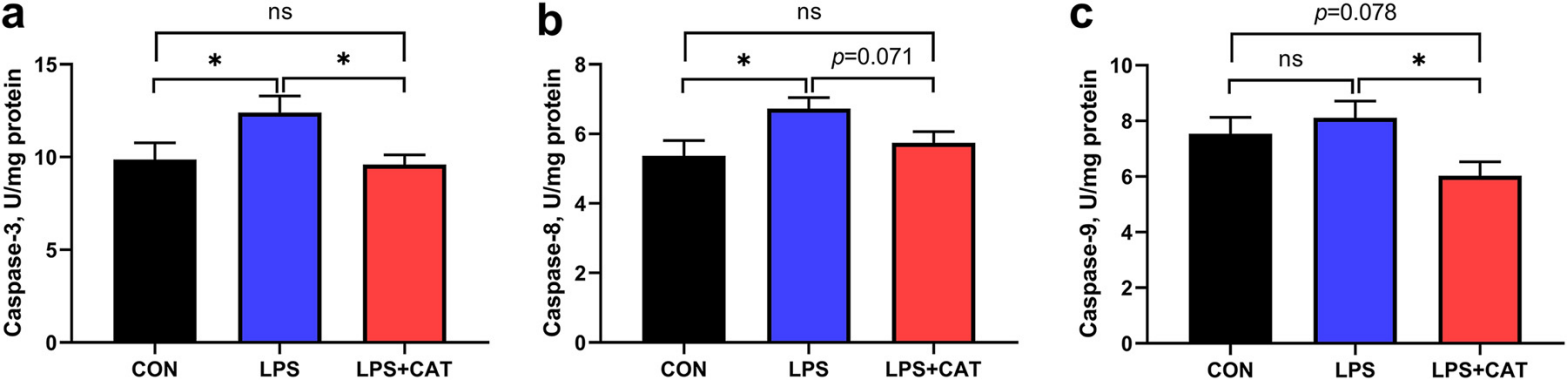
Effects of dietary catalase (CAT) supplementation on caspase activities in jejunal mucosa of weaned pigs challenged with lipopolysaccharide (LPS). (a) Caspase-3; (b) caspase-8; (c) caspase-9. CON, pigs fed the basal diet and given intraperitoneal administration of saline solution; LPS, pigs fed the basal diet and given intraperitoneal administration of LPS; LPS+CAT, pigs fed the basal diet supplemented with 2,000 mg/kg exogenous CAT and given intraperitoneal administration of LPS. The statistical analysis was performed using one-way analysis of variance, and the differences among group means were compared using the least significant difference method. Values are mean ± standard error (*N* = 6). Significant differences are displayed in the figures by * *P* < 0.05, while 0.05 < *P <* 0.10 was considered as a trend to significance. Non-significant differences are indicated by “ns.”

Data for activities of caspase-3, caspase-8, and caspase-9 in jejunal mucosa are summarized in Fig. 5. Relative to the CON pigs, the LPS pigs showed increased caspase-3 and caspase-8 activities (*P < *0.05), and the LPS+CAT pigs had a lower caspase-9 activity (*P = *0.078). In LPS-challenged pigs, dietary supplementation with exogenous CAT markedly reduced the activities of caspase-3, and caspase-9 (*P < *0.05), and had a tendency to decrease the activity of caspase-8 (*P* < 0.071). No significant differences in the activities of caspase-3 and caspase-8 were found between the CON group and the LPS+CAT group (*P > *0.05).

### Gene expression in jejunum mucosa.

**FIG 6:**
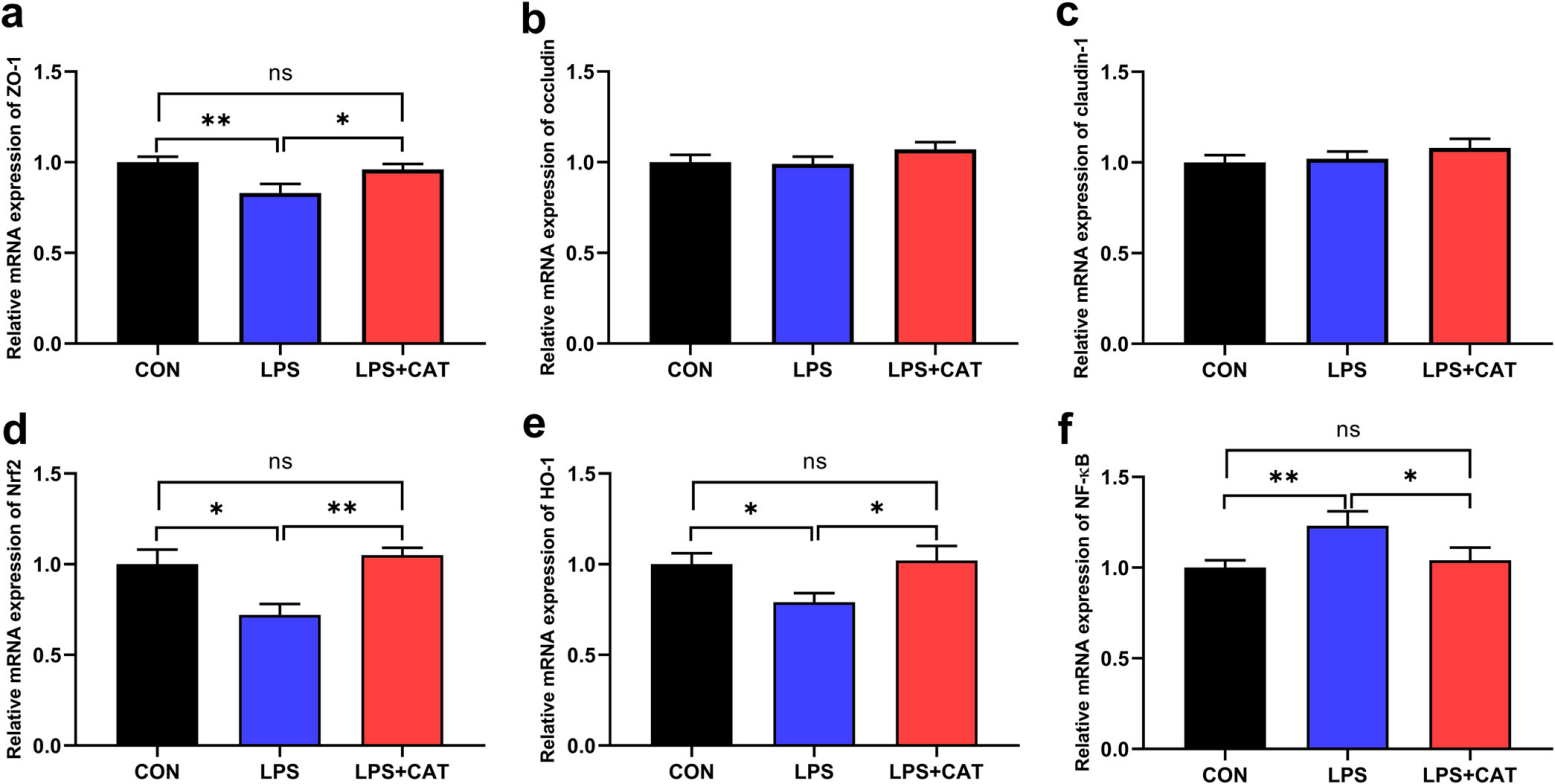
Effects of dietary supplementation with exogenous catalase (CAT) on gene expressions in jejunal mucosa of weaned pigs challenged with lipopolysaccharide (LPS). (a) *ZO-1*; (b) *occludin*; (c) *claudin-1*; (d) *Nrf2*; (e) *HO-1*; (f) *NF-κB*. CON, pigs fed the basal diet and given intraperitoneal administration of saline solution; LPS, pigs fed the basal diet and given intraperitoneal administration of LPS; LPS+CAT, pigs fed the basal diet supplemented with 2,000 mg/kg exogenous CAT and given intraperitoneal administration of LPS. The statistical analysis was performed using one-way analysis of variance, and the differences among group means were compared using the least significant difference method. Values are mean ± standard error (*N* = 6). Significant differences are displayed in the figures by * *P <* 0.05 and ** *P <* 0.01, while 0.05 < *P* < 0.10 was considered as a trend to significance. Nonsignificant differences are indicated by “ns.”

*Relative gene expression of tight junction proteins.* The LPS pigs displayed a decrease (*P <* 0.05) in the mRNA expression of *ZO-1* compared with the CON pigs (Fig. 6a), while dietary supplementation of exogenous CAT upregulated (*P < *0.05) *ZO-1* expression by 15.7% relative to the LPS pigs, which attenuated LPS-induced decrease in *ZO-1* expression (*P > *0.05). There were no significant differences in *occludin* (Fig. 6b) and *claudin-1* (Fig. 6c) relative gene expressions (*P > *0.05).

*Relative gene expression of antioxidant-related genes.* The results revealed that LPS significantly inhibited nuclear factor erythroid 2-related factor (*Nrf2*) (Fig. 6d) and heme oxygenase-1 (*HO-1*) (Fig. 6e) mRNA expression (*P < *0.05), and upregulated *NF-κB* (Fig. 6f) mRNA expression (*P < *0.05) compared with the saline-treated control. In contrast, dietary CAT supplementation significantly upregulated *Nrf2* and *HO-1* expression (*P <* 0.05), and suppressed *NF-κB* expression (*P <* 0.05) against LPS challenge. There were no significant differences in *Nrf2*, *HO-1*, and *NF-κB* mRNA expression between the CON group and the LPS+CAT group (*P > *0.05).

### Microbial diversity in colonic digesta.

**FIG 7:**
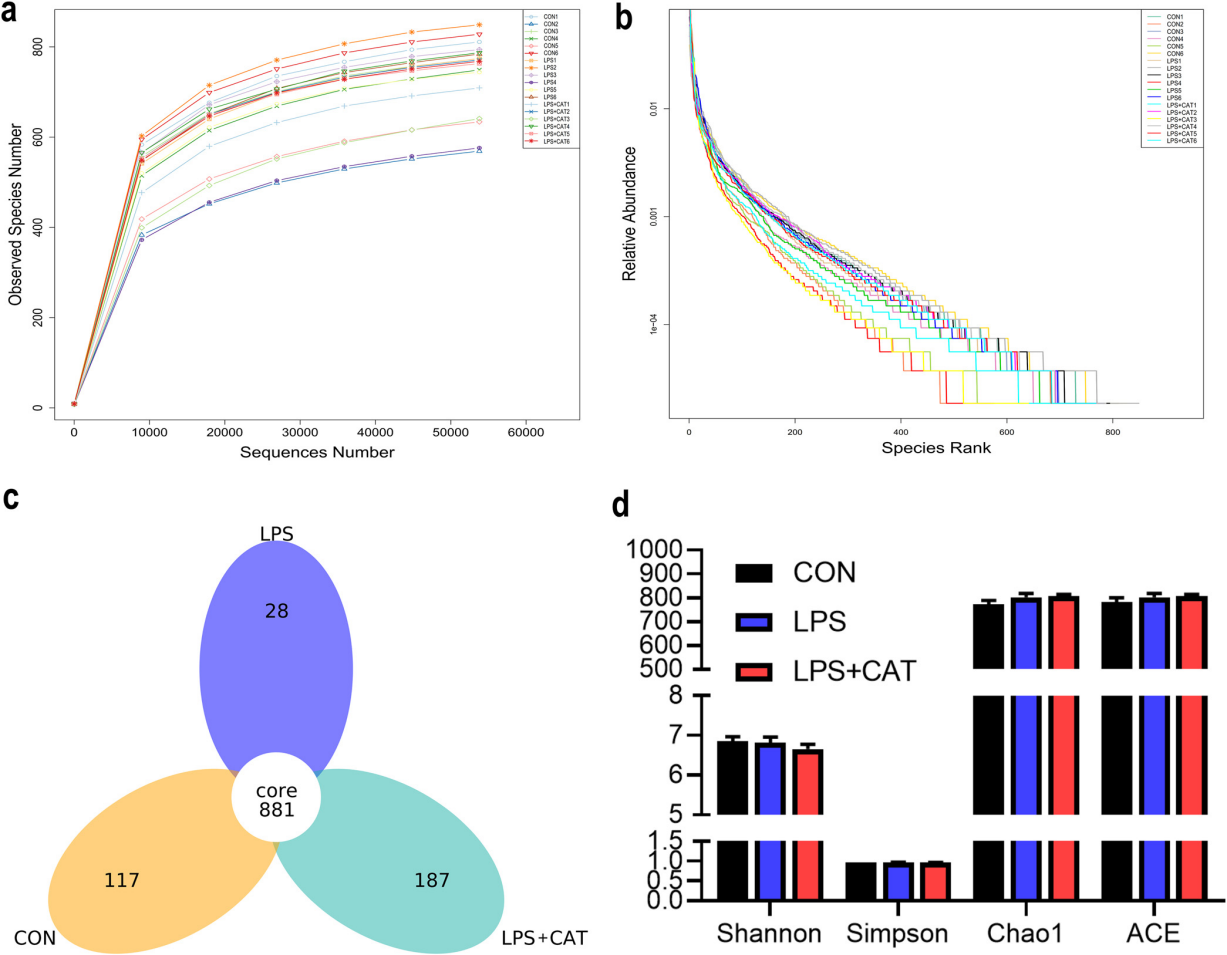
Differences on bacteria community diversity and richness among the three treatments. (a) Rarefaction curve tending to approach the asymptote indicated the sequence depth met the requirements for sequencing and analysis. (b) Rank abundance curve reflected the richness of bacterial community by the width of the curve in the horizontal direction. (c) Stem-and-leaf display was generated to depict shared and unique sequences among the treatments. (d) Alpha diversity indexes, including Shannon, Simpson, Chao 1, and ACE indexes, were used to estimate bacterial community richness and diversity, and values are mean ± standard error (*N* = 6). The statistical differences among the three groups were analyzed using Tukey and Wilcoxon rank-sum tests. CON, pigs fed the basal diet and given intraperitoneal administration of saline solution; LPS, pigs fed the basal diet and given intraperitoneal administration of LPS; LPS+CAT, pigs fed the basal diet supplemented with 2,000 mg/kg exogenous CAT and given intraperitoneal administration of LPS.

*Bacteria community diversity and richness.* Sequence analysis based on the V4 hypervariable region of 16S rDNA gene obtained 1,268,768 total tags from the three groups, including 1,236,628 taxon tags, five unclassified tags, and 32,135 unique tags (Table S1). The taxon tags were clustered into 14,303 bacterial operational taxonomic units (OTUs) using UPARSE pipeline, with an average of 787.00 ± 43.06, 810.00 ± 39.42, and 786.83 ± 31.11 per sample in the CON group, the LPS group, and the LPS+CAT group, respectively. The species accumulation curves (Fig. S1) tended to flatten as the number of analyzed sequences increases up to 18, indicating that our samples were sufficient for OTU testing and could predict the species richness of samples. Rarefaction curve, rank abundance curve, stem and leaf display, and alpha diversity indicators were used to estimate the species richness and diversity. Rarefaction curve (Fig. 7a) based on the OTUs at 97% similarity tended to approach the asymptote, indicating that the sequence depth was also sufficient to represent the majority of species richness and bacterial community diversity. The rank abundance curve (Fig. 7b) suggested that samples from the LPS+CAT group had highest richness of bacterial community in order of LPS+CAT > CON > LPS. Consistently, as shown in the stem-and-leaf display (Fig. 7c), the number of unique sequences was largest (187) in the LPS+CAT group and smallest (28) in LPS group, and 881 in common among the three treatment groups. Results of the microbial alpha diversity indexes including Shannon, Simpson, Chao1, and ACE are shown in Fig. 7d, but no index showed significant difference among the three treatments (*P > *0.05).

**FIG 8:**
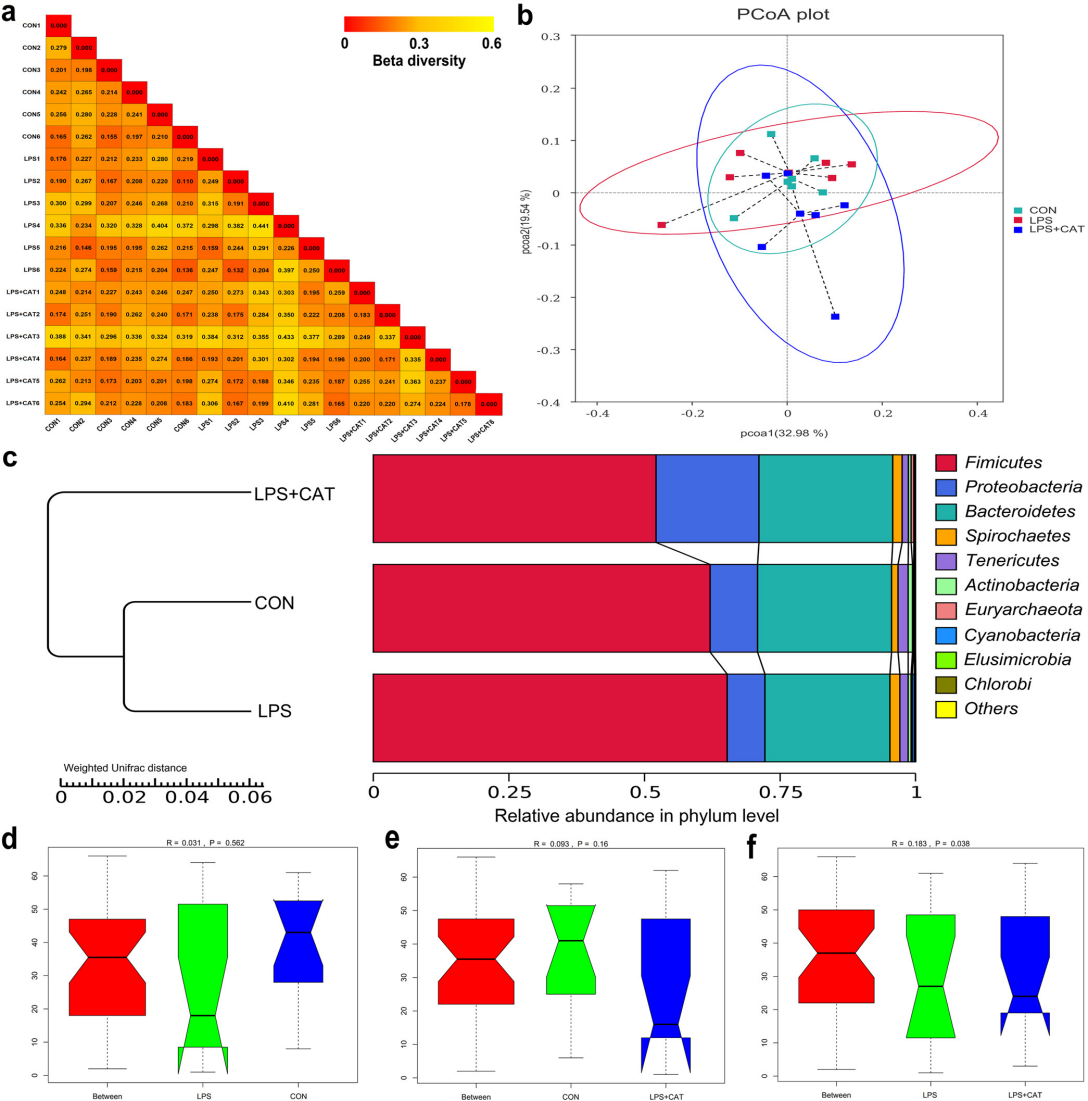
Beta diversity of microbial community analysis. (a) Heat-map of beta diversity for each two samples in all three groups by weighted Unifrac distance. (b) The principal coordinate analysis (PCoA) profile of weighted Unifrac distance. (c) Unweighted pair-group method with arithmetic mean (UPGMA) clustering analysis with weighted Unifrac distance, (d to f) Analysis of ANOSIM (LPS *versus* CON, CON *versus* LPS+CAT, and LPS *versus* LPS+CAT). *R* value is scaled to lie between –1 and +l. Generally, 0 < *R *< 1 and *P <* 0.05 represents that there were significant differences between the groups. CON, pigs fed the basal diet and given intraperitoneal administration of saline solution; LPS, pigs fed the basal diet and given intraperitoneal administration of LPS; LPS+CAT, pigs fed the basal diet supplemented with 2,000 mg/kg exogenous CAT and given intraperitoneal administration of LPS. The statistical differences among the three groups were analyzed using Tukey and Wilcoxon rank-sum tests. *N* = 6.

*Beta diversity of microbial community.* The results of heat-map (Fig. 8a) drawn using the weighted Unifrac distance matric showed that group CON and LPS pairs had the minimum value, while group LPS and LPS+CAT pairs had the maximum value. The principal coordinate analysis (PCoA) profile of weighted Unifrac distance (Fig. 8b) revealed the LPS+CAT samples dispersed far apart with the LPS samples, suggesting a clear separation between the LPS group and the LPS+CAT group. Besides, unweighted pair-group method with arithmetic mean (UPGMA) phylogenetic tree (Fig. 8c) also exhibited that CON group and LPS group had close distance and clustered in one group that indicated the supreme similarity, while the LPS+CAT group distributed in a single branch solely, representing the notably distinguish with the other groups. Analysis of ANOSIM indicated no significant difference (*P > *0.05) was observed in the microbial community structure between group CON and LPS (Fig. 8d) and group CON and LPS+CAT pairs (Fig. 8e), but the LPS group and the LPS+CAT group had remarkably different bacterial community structures (Fig. 8f) (*P* < 0.05).

*Changes of relative abundance in phylum level.* As shown in Fig. 8c, the identified most plentiful phyla were *Firmicutes* and *Bacteroidetes*, accounting for 59.8% and 24.1%, respectively. Of the top 10 phyla, significant differences were observed in the relative abundance of *Firmicutes*, *Proteobacteria*, and *Elusimicrobia* (Fig. S2). Dietary CAT supplementation significantly decreased the *Firmicutes* abundance (*P <* 0.05), and increased the *Proteobacteria* abundance (*P <* 0.05) compared with the CON and LPS groups. The LPS pigs had significantly higher abundance of *Elusimicrobia* than the CON pigs and the LPS+CAT pigs (*P <* 0.05), but there was no significant difference between the CON group and the LPS+CAT group (*P > *0.05).

**FIG 9:**
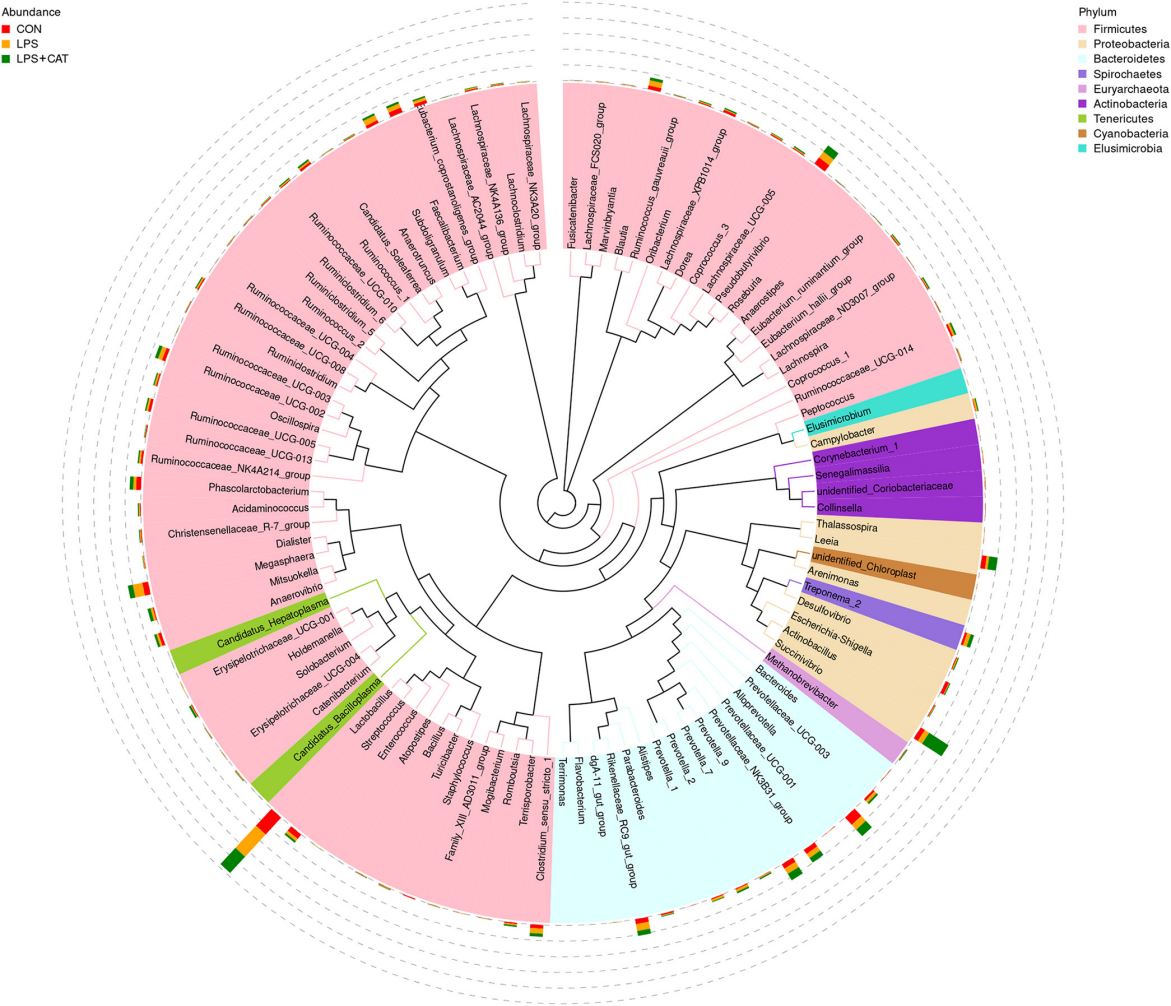
The phylogenetic tree constructed based on the sequence of the top 100 genera. The branches with different colors in the inner circle represent their corresponding phylum, and the stacked column chart in the outer circle indicates the relative abundance of each genus in different treatments. CON, pigs fed the basal diet and given intraperitoneal administration of saline solution; LPS, pigs fed the basal diet and given intraperitoneal administration of LPS; LPS+CAT, pigs fed the basal diet supplemented with 2,000 mg/kg exogenous CAT and given intraperitoneal administration of LPS. *N* = 6.

*Changes of relative abundance in genus level.* The relative abundance at the genera level in the pig colonic microbiota (top 100) is displayed in Fig. 9. The dominate genus among each treatment are presented separately: *Firmicutes* contained 14.8% *Lactobacillus*, 4.76% *Megasphaera*, and 3.48% *Pseudobutyrivibrio*; *Bacteroidetes* included 3.39% *Rikenellaceae*_RC9_gut_group and 3.20% *Alloprevotella* in the CON group. *Firmicutes* composed with 11.2% *Lactobacillus* and 3.78% *Pseudobutyrivibrio*; *Bacteroidetes* included 4.9% *Alloprevotella* in the LPS group; *Proteobacteria* composed of 11.3% *Succinivibrio* and 4.19% *Leeia*; *Firmicutes* contained 10.8% *Lactobacillus* and 3.85% *Pseudobutyrivibrio*; *Bacteroidetes* included 4.31% *Alloprevotella* and 3.73% *Prevotella*_9 in the LPS+CAT group.

**FIG 10:**
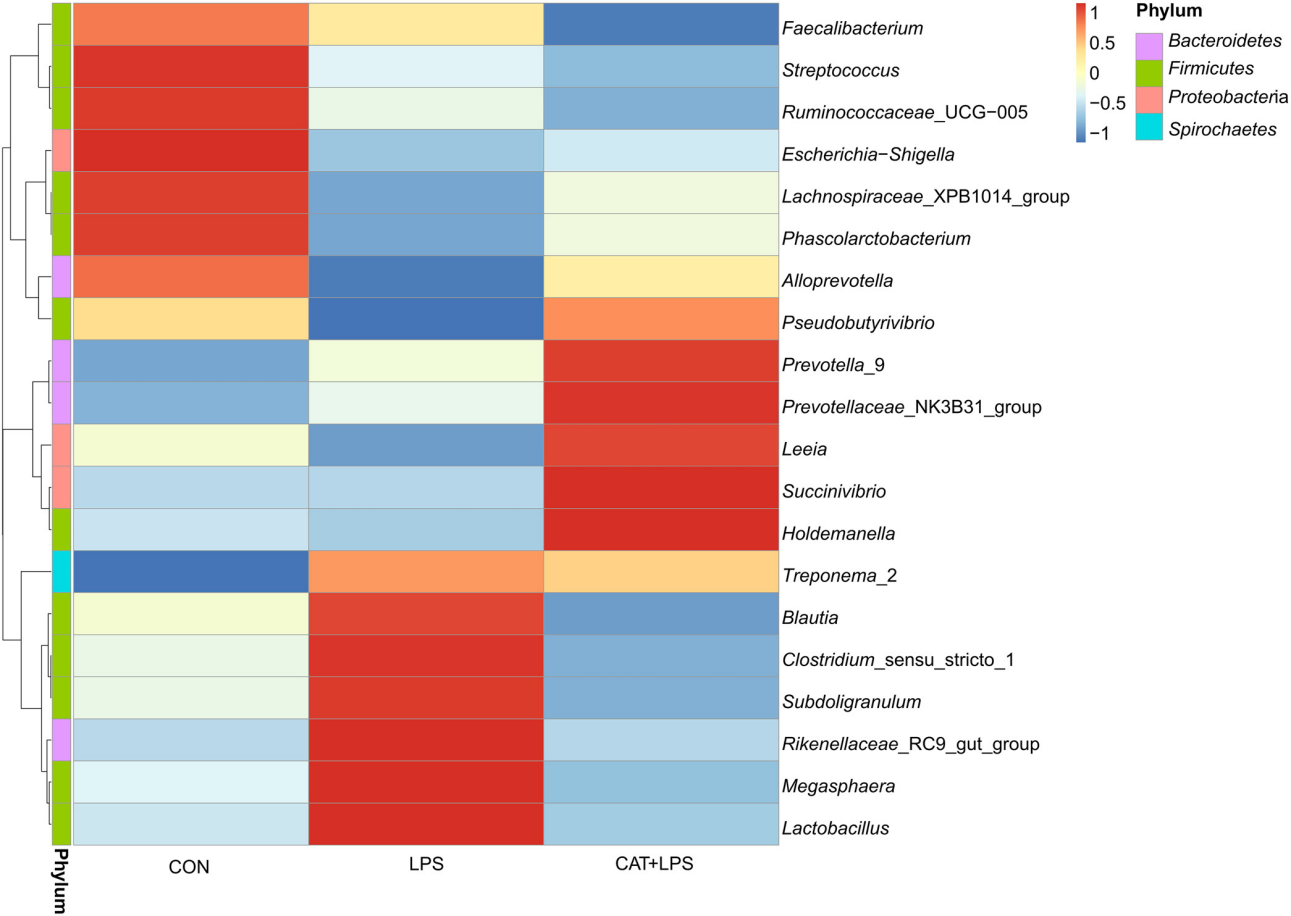
Bacterial community heat-map analysis based on top 20 genera clustering. Different color means the different relative abundance of the genus in all three treatments. CON, pigs fed the basal diet and given intraperitoneal administration of saline solution; LPS, pigs fed the basal diet and given intraperitoneal administration of LPS; LPS+CAT, pigs fed the basal diet supplemented with 2,000 mg/kg exogenous CAT and given intraperitoneal administration of LPS. *N* = 6.

The heat-map (Fig. 10) displayed the species abundance clustering of selected top 20 genera in term of the species annotation and abundance information among all the treatments. Relative to the CON group (Fig. S3), the LPS group showed increased abundance of Streptococcus (*P =* 0.074) and Escherichia-Shigella (*P <* 0.05), and decreased abundance of Subdoligranulum (*P < *0.05). In LPS-challenged pigs, dietary supplementation with exogenous CAT significantly increased (*P* < 0.05) the abundance of Succinivibrio, and markedly reduced (*P <* 0.05) the abundance of Streptococcus, Faecalibacterium, and Escherichia-Shigella. Besides, the LPS+CAT group exhibited notably higher (*P < *0.05) *Succinivibrio* and significantly lower (*P <* 0.05) *Subdoligranulum* and *Faecalibacterium* than the CON group.

## DISCUSSION

Weaning, one of the most crucial developmental periods of the gastrointestinal tract during which diet is transformed from milk to solid feed, is an abruptly stressful event in the pig’s life, which often induces ROS overproduction and disrupts intestinal antioxidant status (5, 17). Weaning-induced oxidative stress could induce weaken intestinal digestion and absorption, destroy intestinal epithelial barrier structure, and change the composition of gut microbiota, resulting in disease, growth retardation, and even death (10, 18). Dietary supplementation plays a critical role in relieving oxidative stress in the initial period after weaning (9, 19, 20). Previous researches indicated that dietary supplementation with exogenous CAT could promote pig growth performance through improving antioxidant capacity of the body and intestinal development (8, 12). Therefore, exogenous CAT is gradually applied to animal production. In the present study, a well-established model of oxidative stress induced by an LPS challenge was employed to investigate the potential protective effects of exogenous CAT on intestinal development and mucosal barrier structure and function in weaned pigs (16, 21).

The small intestine combines the functions of enabling digestion and absorption of selective nutrients and forming a physical barrier to harmful intestinal contents. Efficient uptake of nutrients is executed by the crypt-villus structure. The villus, a finger-like protrusion, extends into the lumen to augment the surface area for nutrient absorption and the crypt is formed by the physically protected epithelial invagination that surrounds the villus base (1). Previous study has reported that the higher villus and shallower crypt contribute to enhancing intestinal absorption, secretion, metabolism, and immune function (19). The ratio of VH to CD is recognized to be a useful criterion to evaluate the nutrient absorption capacity of the small intestine. Therefore, intestinal morphology is an important index of intestinal health (20). Visible intestinal morphological changes, such as villus shedding and atrophy, could be detrimental to the digestion and absorption of nutrients and consequently result in growth retardation (20). The small intestine is highly susceptible to the LPS (16, 21). Previous studies demonstrated that LPS challenge could damage intestinal morphology and trigger intestinal dysfunctions (21, 22). As far as we know, this is the first research to explore whether dietary supplementation with exogenous CAT could alleviate intestinal damage in weaned pigs challenged with LPS. In the present study, LPS administration was found to induce changes of intestinal mucosa morphology, such as diminished VH in jejunum and VCR in ileum and elevated CD in ileum, whereas CAT supplementation resisted the adverse effects of LPS challenge on intestinal morphology, suggesting dietary exogenous CAT supplementation was beneficial to intestinal development in LPS-challenged pigs. Similar results were also found in previous studies that CAT supplementation could enhance the intestinal development and improve the growth of pigs (8, 13). In addition, we found that CAT supplementation could attenuate LPS-induced decrease in the concentrations of DAO and TGF-α to levels observed in saline-treated control. DAO is a cytoplasmic enzyme catalyzing the oxidation of diamines such as histamine, putrescine, and cadaverine, and found in high concentrations in the mature upper villus cells of the intestinal mucosa of humans and other mammalian species (23). Hence, the mucosa DAO activity is regarded as a marker of intestinal mucosal maturation and integrity (24). TGF-α, a potent growth factor belonging to the epidermal growth factor family, was generated by the intestinal epithelial cell and capable of maintaining the integrity of intestinal epithelial cells and protect gastrointestinal mucosa against injury (25). Our previous study also demonstrated that CAT supplementation elevated DAO and TGF-α levels in jejunal mucosa (13). Therefore, CAT supplementation was also conducive to maintaining the maturity and integrity of intestinal epithelial barrier in LPS-challenged pigs. To sum up, dietary CAT supplementation could alleviate LPS-induced intestinal mucosa injury.

Oxidative stress is an important inducing factor in intestinal mucosal damage in weaned pigs (18). In general, enzymatic (represented by CAT, SOD, and GSH-Px) and non-enzymatic (characterized by T-AOC) antioxidant systems maintain a dynamic balance of ROS production and clearance in the body. Endogenously generated ROS, such as superoxide and H_2_O_2_, play a significant role in activating cellular proliferation or survival signaling pathways (11), but oxidative stress happens when ROS generation exceeds the body’s own natural antioxidant defenses, leading to cellular macromolecules damage (9). It was reported that LPS administration could increase acutely ROS production (14). Consistently, in our study, LPS injection elevated the level of H_2_O_2_, and decreased the antioxidant enzymes (CAT and SOD) activities and T-AOC level in intestinal mucosa. Furthermore, we also found that MDA concentration in jejunal mucosa was increased in the LPS pigs. Malondialdehyde concentration is extensively recognized as a reliable parameter for examining the degree of lipid peroxidation, and is closely related to cell damage (26). The results suggested that the antioxidant defense system was destroyed in the LPS-challenged pigs. However, supplementation of 2,000 mg/kg exogenous CAT enhanced the antioxidant enzymes activities, and decreased the levels of MDA and H_2_O_2_ in the jejunal mucosa in the present study. A previous research in mice also demonstrated that dietary supplementation with exogenous CAT could enhance intestinal antioxidant enzymes activities, including SOD, CAT, and GSH-Px, and alleviated high-fat diet-induced intestinal oxidative injury (27). Therefore, it demonstrated that dietary exogenous CAT supplementation could resist LPS-induced intestinal oxidative stress by improving antioxidant enzyme activities of pigs.

A previous study reported that, following inducing the imbalance of redox status, LPS could cause oxidative damage to the intestinal barrier by inducing excessive apoptosis of enterocyte (20). Cell apoptosis is a normal physiological process to eliminate abnormal and damaged cells, but excessive apoptosis inversely results in epithelial cell atrophy, injury, and dysfunction and finally results in the intestinal mucosal barrier injury and gastrointestinal disorders (24, 28). In mammalian cells, the apoptosis response is generally regulated by either the intrinsic pathway or the extrinsic pathway depending upon the origin of stimuli (29), while caspases, a conserved family of enzymes that irreversibly commit a cell to die, are the central components of the apoptotic response (30). The intrinsic pathway is mediated by mitochondria, and several proteins (such as *cytochrome c*) are released from the intermembrane space of mitochondria into the cytoplasm in response to apoptotic stimuli (31), which eventually mediates the activation of caspase-9 (29). The extrinsic pathway is initiated by the binding of an extracellular death ligand to its cell-surface death receptor (such as Fas), and characterized mainly by the activation of caspase-8 (30). Caspase-8 and caspase-9, serving as the initiator caspases, activate the effector caspase (caspase-3) and then initial the process of apoptosis in mammalian cells (29). LPS *in vivo* has been shown to activate caspase-3 which was initiated by caspase-8 or/and caspase-9, and subsequently stimulate apoptosis (8, 32). In the current study, LPS administration was also found to increase caspase-8 and caspase-3 activities, while CAT supplementation decreased caspase-8, caspase-9, and caspase-3 activities in LPS-challenged pigs. The results of the present study suggested that dietary CAT supplementation inhibited excessive apoptosis of enterocyte induced by LPS challenge through suppressing the activation of the intrinsic and extrinsic pathway to protect against mucosa injury. Furthermore, a previous study reported that the decrease of tight junction protein expression is another main reason for oxidative stress-induced intestinal barrier damage (18). ZO-1, a marker of gut permeability, is one of the major tight junction-associated scaffold proteins that binds to transmembrane proteins of tight junctions and the underlying cytoskeleton, and is involved in the regulation of cytoskeletal organization and cell shape (33). In the present study, CAT supplementation increased mRNA expression of *ZO-1* in the jejunal mucosa of LPS-challenged pigs. Therefore, these results indicated that dietary CAT supplementation could improve the intestinal barrier function partly by preventing cell apoptosis and upregulating tight junction protein expression, which might be the partial reasons why dietary exogenous CAT could attenuate oxidative stress-impaired intestinal permeability in weaned pigs.

To identify possible mechanisms of dietary catalase in relieving oxidative stress, we quantified the gene expression levels of *Nrf2* and *HO-1* in intestinal mucosa. Previous studies have confirmed that the Nrf2/HO-1 pathway plays a critical role in antioxidative stress (26, 34). Nrf2 is one of the most important transcription factors in eukaryotic cells, and regulates the expression of an array of enzymes with important antioxidant and detoxifying functions (35). Thus, Nrf2 is regarded as one of the initiating mechanisms of oxidative stress. Many studies have shown that when oxidative stress occurs, Nrf2 is activated and then upregulates the expression of its downstream target HO-1, an important antioxidant enzyme regulating the ROS levels in cells, to protect against oxidative stress (34). It was reported that Nrf2/HO-1 signaling pathway was often suppressed in LPS-challenged pigs (36). Our current study showed that CAT supplementation significantly upregulated *Nrf2* and *HO-1* expression in LPS-challenged weaned pigs, which was in line with the improved effect of CAT on antioxidant enzymes activities mentioned above, indicating that dietary supplementation with 2,000 mg/kg CAT enhanced intestinal antioxidant capacity of the weaned pigs challenged by LPS and might improve the antioxidant enzymes activities through activating the *Nrf2/HO-1* pathway.

Accumulating evidence has confirmed that oxidative stress that induced the overproduction of ROS could also damage the intestinal barrier by inducing inflammatory response (14, 18). NF-κB signaling pathway is considered as a prototypical proinflammatory signaling pathway to regulate stress responses, apoptosis, immunity, and differentiation (36). A previous study indicated that the LPS-generated ROS could activate NF-κB at both the mRNA and protein level (15). Accordingly, we found that LPS injection increased TNF-α and IL-6 concentrations as well as NF-κB relative mRNA expression in jejunum mucosa in the present study. TNF-α, a major proinflammatory cytokine produced by activated macrophages, can induce the NF-κB pathway activation (36), while IL-6, secreted from various types of cells, such as T-cells, B-cells, and endothelial cells, can contribute to the pathogenesis of inflammatory diseases (37). In addition, IL-6 production can also be stimulated by TNF-α signaling (38). Thus, it is believed that excessive production of cytokines such as TNF-α and IL-6 represented an important etiologic factor in LPS-induced intestinal damage (22). In the present study, dietary CAT supplementation attenuated LPS-induced increase in intestinal mucosal concentrations of TNF-α and IL-6 to levels observed in saline-treated control, suggesting that CAT could relieve LPS-induced intestinal mucosa damage in weaned pigs by inhibiting the inflammatory response. This result further indicated the improved effect of dietary exogenous CAT on antioxidant capacity in weaned pigs. Secretory immunoglobulin A, acting as one of the major immunological mechanisms of defense against the potential attack of pathogens, is secreted by intestinal lamina propria plasmacytes and plays a central role in mucosal immunity (39). A previous study showed that the content of SIgA in the intestine was significantly decreased after LPS treatment (40). In the present study, decreased SIgA concentration was also found to be decreased by LPS challenge, but dietary supplementation with exogenous CAT alleviated LPS-induced decrease in SIgA concentration, which suggests that supplementation of exogenous CAT enhanced the capacity of weaned pigs to resist the invasion of pathogenic bacteria.

The intestinal mucosa is a cellular barrier and the main site of interaction with foreign substances and exogenous microorganisms, and a mature mucosal barrier serves as a primary innate defense against pathogens (41). Nevertheless, increased harmful bacteria will damage the integrity of intestinal mucosa and lead to intestinal barrier dysfunction (42). In the present study, high-throughput sequencing showed that pigs in LPS+CAT group had an obviously distinct microbial community structures from those in LPS group though no significant differences were observed in alpha diversity indexes. Xu et al. (2020) also showed that LPS administration after 4 h had no significant effects on ACE, Chao 1, and Shannon indexes in colonic digesta in weaned pigs (43), which might be because it took time for gut microbiota to change. Meanwhile, a previous study demonstrated that CAT supplementation did not affect alpha diversity indexes, but changed the beta diversity of feces in weaned pigs (13). The result that no significant difference was observed in the microbial community structure between group CON and group LPS+CAT suggests that CAT supplementation could resist the invasion of pathogenic bacteria. Consistently, CAT supplementation inhibited the proliferation of Elusimicrobia, Streptococcus, and Escherichia-Shigella induced by LPS challenge. *Elusimicrobia* was usually used as a potential biomarker of intestinal injury, and a potential negative effect on health (44), while Streptococcus and Escherichia-Shigella are common pathogenic bacteria related to numerous inflammatory responses (45, 46). A previous study also found that dietary CAT supplementation decreased the fecal abundances of Streptococcus and Escherichia-Shigella in weaned pigs (13). It indicated that supplementation of CAT could inhibit LPS-induced proliferation of pathogenic bacteria. Another intriguing discovery in the current study was that dietary supplemented with CAT obviously decreased *Firmicutes* (characterized by *Faecalibacterium*) and increased *Proteobacteria* (represented by *Succinivibrio*) compared with the other two treatments. *Firmicutes*, *Proteobacteria*, and *Bacteroides* were the most abundant phyla in all groups, which was in accordance with previous studies (13, 24). In general, chronic inflammatory response was associated with an altered gut microbiota characterized by elevated levels of *Firmicutes* (47). *Succinivibrio*, which contributed to the increase of *Proteobacteria* in LPS+CAT pigs, plays a role in cellulose and hemicellulose degradation and metabolites of degradation have been shown to inhibit intestinal colonization by pathogenic bacteria (48). It was reported that *Succinivibrio* was positively correlated with IL-17 and interferon-γ (IFN-γ), two multifaceted cytokines with diverse roles in immune protection (49). Previous studies also showed that CAT addition in pig’s diet altered the structure of intestinal flora and decreased the abundance of harmful bacteria (13, 34). Gut microflora has been proven to play a major role against exogenous bacteria through colonization resistance (41). Our results illuminated that CAT supplementation could provide resistance against colonization and invasion by pathogens through forming a stable microbial community composition. Above all, CAT supplementation promoted the proliferation of beneficial bacteria and protected against LPS-induced proliferation of harmful bacteria, which might be another reason for CAT alleviating mucosa injury induced by LPS challenge in the present study.

In conclusion, oxidative stress induced by LPS administration damaged intestinal mucosa morphology and barrier through decreasing intestinal antioxidant capacity, triggering inflammatory response, promoting enterocyte apoptosis, and disturbing intestinal flora structure of weaned pigs. Supplementation of 2,000 mg/kg CAT is conducive to improve intestinal development and function, and protect the intestinal mucosa against LPS-induced injury in weaned pigs. The beneficial effects of dietary CAT supplementation are due in part to the enhancement of intestinal antioxidant capacity and change of gut microbiota composition in weaned pigs. These findings suggested that CAT, acting as an antioxidant, is potentially an effective feed additive for protecting weaned pigs against weaning stress-induced intestinal damage. Moreover, this study will also assist in the developing of CAT produced by microorganisms to attenuate various oxidative stress-induced injury or diseases.

## MATERIALS AND METHODS

### Ethical approval.

The present research was conducted at the Animal Husbandry Science and Technology Park of Shandong Agricultural University, Tai’an, China. The animal protocol for this research was approved by the Care and Use committee of Shandong Agricultural University (Approval Number: SDAUA-2019-019).

### Animals and treatments.

Fifty-four Duroc × Landrace × Yorkshire crossbred weaned pigs, with an initial average body weight (BW) of 6.90 ± 0.01 kg and weaned at 21 days, were randomly allocated to one of three dietary treatments for a 35-day study. The three experimental treatments were as follows: (i) nonchallenged control (CON group, pigs received a basal diet and given intraperitoneal administration of sterile solution); (ii) LPS-challenged control (LPS group, pigs received the basal diet and given intraperitoneal administration of LPS); and (iii) LPS + 2,000 mg/kg CAT (LPS+CAT group, pigs received the basal diet with 2,000 mg/kg exogenous CAT supplementation and given intraperitoneal administration of LPS). The 54 pigs were housed in nine pens located in a room with three females and three males per pen (1.5 × 2.5 m) and three pens per treatment.

### Diets and management.

The present study was conducted with corn-soybean meal basal diets, and a two-phase feeding program (phase I, 1 to 21 days; phase II, 22 to 35 days) was used to meet nutrient requirements at different growth stages in this experiment. The two basal diets (Table S2) were formulated in line with the National Research Council’s (2012) recommendation for weaned pigs. The CAT production (Liaoning Vetland Bio-Technology Co., Ltd, Liaoning, China), with an activity of 60 U/g, was produced by *Penicillium notatum* which was available at the Agricultural Culture Collection of China (ACCC) under the accession number ACCC 30443. After spray drying and sieving, the CAT was added to the basal diets by replacing equal amount of corn. Pigs were kept in an entirely enclosed room equipped with a temperature-controlled system. Pigs were fed four times per day (0800, 1200, 1600, and 2000 h), and had free access to diets and water during the whole process of experiment. The ambient temperature was controlled at 25 to 28°C. No antibiotics or vaccines were given to the pigs during the study.

In total, 18 overnight fasted pigs (two healthy pigs per pen [one female and one male] with BWs closest to the average pen weight) were selected from the three groups after the 35-day feeding. The pigs in groups LPS and LPS+CAT were intraperitoneally administered with LPS (50 μg/kg BW; Escherichia coli L2880; Sigma-Aldrich, St. Louis, MO, USA) which was dissolved in 0.9% NaCl solution to a concentration of 400 mg/L before administration (17), whereas the pigs in the CON group received intraperitoneal injection of an equal amount of 0.9% NaCl solution. All the selected pigs were kept in individual metabolism cages measuring 1.8 m^2^ × 0.7 m^2^ and had free access to diets and water. Measurement of rectal temperatures of each pig was taken 0 h and 4 h after saline or LPS administration to make sure that LPS challenge model was established successfully (Fig. S4).

### Sample collections.

On day 36, all 18 pigs were immediately slaughtered for sampling under intramuscular anesthesia with Zoletile 50 Vet (Virbac, Carros, France; 0.1 mg/kg BW) 4 h after LPS or saline administration (8). After the abdomen was incised, the entire small intestine, the segments of the alimentary tract between the pylorus and the ileocecal junction, was removed and divided into three parts: duodenum, jejunum, and ileum as described in Li et al. (13). Segments of 2 cm length cut from the medium of the duodenum, jejunum, and ileum were flushed gently by saline solution and fixed in 4% paraformaldehyde for further analysis. After being opened along with mesentery, the remaining part of the jejunum was washed in ice-cold saline solution and subsequently scraped carefully with a sterile glass slide to collect mucosa tissue. The scraped mucosal tissue was chilled in liquid nitrogen and subsequently preserved at −80°C until analysis. Furthermore, the colonic contents were quickly collected as previously described (26), and stored at −80°C for microbiological analysis.

### Intestinal morphology measurements.

The intestinal segments were removed from the paraformaldehyde solution, dehydrated with normal saline, and embedded according to routine paraffin-embedding protocol after a 24-h fixation (24), and processed into serial 5-μm sections using a Leica semiautomatic microtome, followed by straining with hematoxylin and eosin and sealing with a neutral resin size. Photomicrographs were obtained using an Olympus BX51 digital microscope (Olympus, Tokyo, Japan) under 100× magnification. VH and CD were measured with JD801 morphologic image analysis software (JEDA, Nanjing, Jiangsu, China) as described previously (13). Briefly, a total of 12 to 20 integrated, well oriented crypt-villus units were randomly chosen and quantified in each sample. The VH was measured from the tip of the villus to the base between individual villus, and the CD measurements were taken from the valley between individual villus to the basal membrane. The ratio of VH to CD, defined as VCR, was calculated as the VH divided by CD.

### Determination of intestinal mucosal barrier integrity and SIgA concentration.

The determination of TFF, TGF-α, DAO activities, MCH-II, and SIgA in jejunal mucosa were proceeded with the specific ELISA kits purchased from Beijing winter song Boye Biotechnology Co. Ltd., Beijing, China (19, 24). First, the jejunal mucosa samples were homogenized in ice-cold saline solution (1:9, wt/vol), and the supernatants were obtained after centrifugation at 12,000 × *g* for 10 min at 4°C. Next, 50 μL of the prepared supernatant and diluted standard solutions was added to the corresponding microplates. After a 30-min incubation at 37°C, the microplates were washed five times with diluted wash solution and 50 μL of the HRP-Conjugated Reagent was added to each well. Chromogenic procedure was performed with two kinds of chromogenic agents for 15 min at 37°C followed by Stop Buffer addition. Finally, the absorbance of each well was read within 15 min at 450 nm, and the levels of DAO, TFF, TGF-α, MCH-II, and SIgA were calculated using the standard curve made with standard solutions. All samples were determined in duplicate.

### Determination of intestinal oxidant and antioxidant parameters.

Jejunal mucosa oxidant and antioxidant parameters contained CAT, SOD, T-AOC GSH-Px, MDA, and H_2_O_2_. The parameters, including CAT, SOD, GSH-Px, T-AOC, and MDA, were examined with specific assay kits purchased from Nanjing Jiancheng Bioengineering Institute (Nanjing, China) as previously described (8). Likewise, the supernatants of jejunal mucosa were prepared. The CAT activity was determined by monitoring the rate at which it caused the breakdown of H_2_O_2_ at 240 nm. The SOD activity was measured with the WST-1 method, using a xanthine/xanthine oxidase system to generate O_2_^2−^. The contained WST-1 formed a water-soluble formazan dye when reduced by O_2_^2−^, and the inhibition of WST-1 reduction was used to measure SOD activity at 450 nm. The activity of GSH-Px was calculated by measuring the rate at which it catalyzed the reaction of glutathione with H_2_O_2_ at 412 nm. The T-AOC level was examined by the reduction in Fe^3+^-tripyridyltriazine to Fe^2+^-tripyridyltriazine at 520 nm. The MDA concentration was measured by reaction with thiobarbituric acid at 95°C, and the produced red compounds had absorption peak at 532 nm. The levels of CAT, SOD, GSH-Px, T-AOC, and MDA were normalized to each sample’s total protein concentration measured by the Braford method. The H_2_O_2_ level of jejunal mucosa was assessed with the commercial kits (Beyotime Biotech, Shanghai, China) as described in detail in our previous study (13).

### Determination of intestinal inflammatory cytokines concentrations.

The concentrations of TNF-α and IL-6 in jejunal mucosa were examined strictly in according with the respective instructions of ELISA kits (R&D Systems Inc., Minneapolis, MN, USA). The detection steps are as follows. In brief, 100 μL of supernatant of tissue homogenate or standards were added to the prepared microplates, and incubated 2 h at room temperature. The microplates were washed with 400 μL of Wash Buffer, and 100 μL of the detection antibody was added to each well, followed by incubation for 2 h at room temperature. After being washed again, the wells were added with 100 μL of the working dilution of Streptavidin-HRP, and incubated for 20 min at room temperature. Finally, the optical density of each well was determined immediately using a microplate reader set at 450 nm, following addition of 100 μL of substrate solution and 50 μL of stop solution to each well in order. The concentrations of TNF-α and IL-6 in jejunal mucosa were calculated using the standard curve made with standard solutions and normalized to each sample’s total protein concentration.

### Determination of intestinal mucosa caspase activities.

Jejunal mucosa caspase-3, caspase-8, and caspase-9 activities were assessed with a commercial ELISA kits (Beyotime Biotech) according to the manufacturer’s instruction as described in Wan et al. (20). Briefly, the jejunal mucosa samples (100 mg) were homogenized in 1 mL of lysis buffer, and placed in an ice bath for 5 min. After centrifugation at 20,000 × *g* for 10 min at 4°C, the supernatants of jejunal mucosal tissues were obtained. At the same time, the caspase substrates of caspase-3, caspase-8, and caspase-9 (Ac-DEVD-*p*NA, Ac-IETD-*p*NA, and Ac-LEHD-*p*NA, respectively) were prepared. Then, 10 μL of each caspase substrate and 40 μL of reaction buffer were added into microplates successively, followed by incubation for 60 to 120 min at 37°C in a water bath. The absorbance of the mixtures was measured at 405 nm when a noticeable color change was observed. The activities of caspases in jejunal mucosa were normalized to each sample’s total protein concentration.

### Gene expression analysis.

Total RNA was extracted from the jejunal mucosa sample with TRIzol reagent (Invitrogen, Carlsbad, CA, USA) in terms of the manufacturer’s instructions. The cDNA was synthesized with the TaKaRa reverse transcription kit (TaKaRa Biotechnology, Tokyo, Japan) following RNA quality and concentration measurement. The mRNA expression levels of *Nrf2*, *HO-1*, *NF-κB*, *ZO-1*, *occludin*, and *claudin-1* in jejunal mucosa tissues were assessed using an ABI 7900HT fast real-time PCR system (Applied Biosystems, Foster City, CA, USA) with SYBR green real-time PCR regent (TaKaRa Biotechnology, Tokyo, Japan) as previously described (26). Primer sequences are shown in Table S3. Glyceraldehyde-3-phosphate dehydrogenase (*GAPDH*) was used as the housekeeping gene for gene normalization and quantification. The relative mRNA abundances of the target genes were calculated using the threshold cycle (2^-ΔΔCt^) method.

### Bioinformatic analysis.

The bacterial genomic DNA was extracted from frozen colonic contents of the 18 slaughtered weaned pigs (six pigs per treatment) with an E.Z.N.A. ^TM^ Stool DNA kit (Omega Bio-Tek, Norcross, GA, USA) according to the manufacturer’s protocol. Briefly, the instructions are to weigh up to 50 mg to 100 mg of frozen colonic contents in a 2 mL centrifuge tube containing 200 mg of glass beads and place on ice, then add 300 μL of Buffer SP1, 10 μL of Proteinase K, and 100 μL of Buffer SP2. After centrifugation at 13,000 × *g* in a microcentrifuge for 5 min, the supernatant was transferred to a new 1.5 mL microfuge tube, and 200 μL of HTR reagent was added into the tube. Then, centrifuge again and transfer 250 μL supernatant to a new 2.0 mL tube, following incubation at room temperature for 2 min. Finally, 250 μL of BL buffer was added into the tube, and the genomic DNA was obtained after centrifugation for 2 min at room temperature to dry the Hibind DNA column. The extracted DNA was monitored on 1% agarose gels for DNA concentration and purity examination, and diluted to 1 ng/μL using sterile water. Then, the V4 hypervariable region of 16S rDNA was amplified with 515F and 806R primer (5′-GTGCCAGCMGCCGCGGTAA-3′ and 5′-GGACTACHVGGGTWTCTAAT-3′, respectively) (24), and the PCR products was purified with Qiagen Gel extraction kit (Qiagen, Germany). Subsequently, sequencing libraries were generated, and the library quality was measured using a Qubit Fluorometer (Thermo Fisher Scientific, United Kingdom) and Agilent Bioanalyzer 2100 system (Agilent Technologies, CA, USA). Finally, the library was sequenced on the Illumina NovaSeq platform (Novogene, Beijing, China), and 250 bp paired-end reads were generated. Negative controls were used in each round of amplification to identify the sterility of reagents, and a mock microbial community bacterial community was included in the sequencing run as a control to assess error rate and inter-run variability. After paired-end reads assembly, data filtration, and chimera removal, the effective sequences were finally obtained. The high-quality sequences sharing over 97% sequence similarity were clustered into the same OTUs using UPARSE pipeline (50), and then classified to different taxonomic levels using SILVA database based on Mothur algorithm to annotate taxonomic information (51). Alpha diversity (including Shannon, Simpson, Chao 1, and ACE indexes) and weighted Unifrac distance were performed in the Quantitative Insights Into Microbial Ecology (QIIME, V1.7.0) and exhibited with R software (Version 3.1) (52). The dissimilarity matrices of OTUs were visualized using PCoA and UPGMA phylogenetic tree (53). The statistical differences in alpha and beta diversity of bacterial communities among the three groups were analyzed using Tukey and Wilcoxon rank-sum tests. Significant differences among the microbial communities were detected with the analysis of similarity (ANOSIM) test.

### Statistical analysis.

The pig was considered as the experimental unit for all the variables. First of all, all indices were analyzed as a 2 × 2 factorial arrangement (treatment and sex) to evaluate the effect of sex and the interaction between treatment and sex, and no effects of sex and interaction between treatment and sex were observed in the present study. Then, statistical analysis was performed using one-way analysis of variance (ANOVA) of SAS 9.4 (Institute Inc., Cary, NC, USA) after data were assessed for normal distribution using Shapiro-Wilk’s statistic (W > 0.05). The data were plotted in the figures as mean ± standard error. The differences among group means were compared using the least significant difference method. Significant differences are displayed in the figures by * *P < *0.05, ** *P* < 0.01, and *** *P* < 0.001, while 0.05 < *P < *0.10 was considered as a trend to significance. Nonsignificant differences are indicated by “ns.”

### Data availability.

All sequencing data are deposited in the Sequence Read Archive of the National Center for Biotechnology Information under accession PRJNA560659 (Illumina sequences).

## References

[1] Beumer J, Clevers H. 2021. Cell fate specification and differentiation in the adult mammalian intestine. Nat Rev Mol Cell Biol 22:39–53. doi:10.1038/s41580-020-0278-0. 32958874

[2] Taleb S. 2019. Tryptophan dietary impacts gut barrier and metabolic diseases. Front Immunol 10:2113. doi:10.3389/fimmu.2019.02113. 31552046 PMC6746884

[3] González-González M, Díaz-Zepeda C, Eyzaguirre-Velásquez J, González-Arancibia C, Bravo JA, Julio-Pieper M. 2018. Investigating gut permeability in animal models of disease. Front Physiol 9:1962. doi:10.3389/fphys.2018.01962. 30697168 PMC6341294

[4] Yin J, Wu MM, Xiao H, Ren WK, Duan JL, Yang G, Li TJ, Yin YL. 2014. Development of an antioxidant system after early weaning in piglets. J Anim Sci 92:612–619. doi:10.2527/jas.2013-6986. 24352957

[5] Campbell JM, Crenshaw JD, Polo J. 2013. The biological stress of early weaned piglets. J Anim Sci Biotechnol 4:19. doi:10.1186/2049-1891-4-19. 23631414 PMC3651348

[6] Liu Y, Bao Z, Xu X, Chao H, Lin C, Li Z, Liu Y, Wang X, You Y, Liu N, Ji J. 2017. Extracellular signal-regulated kinase/nuclear factor-erythroid2-like2/heme oxygenase-1 pathway-mediated mitophagy alleviates traumatic brain injury-induced intestinal mucosa damage and epithelial barrier dysfunction. J Neurotrauma 34:2119–2131. doi:10.1089/neu.2016.4764. 28093052

[7] Figueira TR, Barros MH, Camargo AA, Castilho RF, Ferreira JC, Kowaltowski AJ, Sluse FE, Souza-Pinto NC, Vercesi AE. 2013. Mitochondria as a source of reactive oxygen and nitrogen species, from molecular mechanisms to human health. Antioxid Redox Signal 18:2029–2074. doi:10.1089/ars.2012.4729. 23244576

[8] Li Y, Zhao X, Jiang X, Chen L, Hong L, Zhuo Y, Lin Y, Fang Z, Che L, Feng B, Xu S, Li J, Wu D. 2020. Effects of dietary supplementation with exogenous catalase on growth performance; oxidative stress; and hepatic apoptosis in weaned piglets challenged with lipopolysaccharide. J Anim Sci 98:skaa067. doi:10.1093/jas/skaa067. 32152634 PMC7205395

[9] Degroote J, Vergauwen H, Van Noten N, Wang W, De Smet S, Van Ginneken C, Michiels J. 2019. The effect of dietary quercetin on the glutathione redox system and small intestinal functionality of weaned piglets. Antioxidants 8:312. doi:10.3390/antiox8080312. 31426309 PMC6720349

[10] Hu CH, Xiao K, Luan ZS, Song J. 2013. Early weaning increases intestinal permeability; alters expression of cytokine and tight junction proteins; and activates mitogen-activated protein kinases in pigs. J Anim Sci 91:1094–1101. doi:10.2527/jas.2012-5796. 23230104

[11] Kajarabille N, Latunde-Dada GO. 2019. Programmed cell-death by ferroptosis, antioxidants as mitigators. Int J Mol Sci 20:4968. doi:10.3390/ijms20194968. 31597407 PMC6801403

[12] Chen J, Zhang Z, Zhan X, Xia M, Liu Z, Dong T, Tian J, Ye H, Feng D, Zuo J. 2019. Effects of catalase supplementation in diets of sows on growth performance and antioxidant capacity of sows and piglets. Chin J Anim Nutr 31:3268–3275.

[13] Li Y, Zhao X, Zhang L, Zhan X, Liu Z, Zhuo Y, Lin Y, Fang Z, Che L, Feng B, Xu S, Li J, Wu D. 2020. Effects of a diet supplemented with exogenous catalase from *Penicillium notatum* on intestinal development and microbiota in weaned piglets. Microorganisms 8:391. doi:10.3390/microorganisms8030391. 32168962 PMC7143822

[14] Gatto F, Moglianetti M, Pompa PP, Bardi G. 2018. Platinum nanoparticles decrease reactive oxygen species and modulate gene expression without alteration of immune responses in THP-1 monocytes. Nanomaterials (Basel) 8:392. doi:10.3390/nano8060392. 29865145 PMC6027382

[15] Shah SA, Khan M, Jo MH, Jo MG, Amin FU, Kim MO. 2017. Melatonin stimulates the sirt1/nrf2 signaling pathway counteracting lipopolysaccharide (LPS)-induced oxidative stress to rescue postnatal rat brain. CNS Neurosci Ther 23:33–44. doi:10.1111/cns.12588. 27421686 PMC6492734

[16] Zhu C, Wu Y, Jiang Z, Zheng C, Wang L, Yang X, Ma X, Gao K, Hu Y. 2015. Dietary soy isoflavone attenuated growth performance and intestinal barrier functions in weaned piglets challenged with lipopolysaccharide. Int Immunopharmacol 28:288–294. doi:10.1016/j.intimp.2015.04.054. 25979760

[17] Luo Z, Zhu W, Guo Q, Luo W, Zhang J, Xu W, Xu J. 2016. Weaning induced hepatic oxidative stress; apoptosis; and aminotransferases through MAPK signaling pathways in piglets. Oxid Med Cell Longev 2016:4768541. doi:10.1155/2016/4768541. 27807471 PMC5078666

[18] Cao ST, Wang CC, Wu H, Zhang QH, Jiao LF, Hu CH. 2018. Weaning disrupts intestinal antioxidant status, impairs intestinal barrier and mitochondrial function, and triggers mitophagy in piglets. J Anim Sci 96:1073–1083. doi:10.1093/jas/skx062. 29617867 PMC6093500

[19] Chen J, Xie H, Chen D, Yu B, Mao X, Zheng P, Yu J, Luo Y, Luo J, He J. 2018. Chlorogenic acid improves intestinal development via suppressing mucosa inflammation and cell apoptosis in weaned pigs. ACS Omega 3:2211–2219. doi:10.1021/acsomega.7b01971. 30023826 PMC6044628

[20] Wan J, Zhang J, Chen D, Yu B, Mao X, Zheng P, Yu J, Huang Z, Luo J, Luo Y, He J. 2018. Alginate oligosaccharide alleviates enterotoxigenic *Escherichia coli*-induced intestinal mucosal disruption in weaned pigs. Food Funct 9:6401–6413. doi:10.1039/C8FO01551A. 30457630

[21] Chen Y, Mou D, Hu L, Zhen J, Che L, Fang Z, Xu S, Lin Y, Feng B, Li J, Wu D. 2017. Effects of maternal low-energy diet during gestation on intestinal morphology, disaccharidase activity, and immune response to lipopolysaccharide challenge in pig offspring. Nutrients 9:1115. doi:10.3390/nu9101115. 29027951 PMC5691731

[22] Chen L, Li S, Zheng J, Li W, Jiang X, Zhao X, Li J, Che L, Lin Y, Xu S, Feng B, Fang Z, Wu D. 2018. Effects of dietary *Clostridium butyricum* supplementation on growth performance, intestinal development, and immune response of weaned piglets challenged with lipopolysaccharide. J Anim Sci Biotechnol 9:62. doi:10.1186/s40104-018-0275-8. 30159141 PMC6106813

[23] Schnedl WJ, Schenk M, Lackner S, Enko D, Mangge H, Forster F. 2019. Diamine oxidase supplementation improves symptoms in patients with histamine intolerance. Food Sci Biotechnol 28:1779–1784. doi:10.1007/s10068-019-00627-3. 31807350 PMC6859183

[24] Chen J, Yu B, Chen D, Zheng P, Luo Y, Huang Z, Luo J, Mao X, Yu J, He J. 2019. Changes of porcine gut microbiota in response to dietary chlorogenic acid supplementation. Appl Microbiol Biotechnol 103:8157–8168. doi:10.1007/s00253-019-10025-8. 31401751

[25] McEntee CP, Gunaltay S, Travis MA. 2020. Regulation of barrier immunity and homeostasis by integrin-mediated transforming growth factor β activation. Immunology 160:139–148. doi:10.1111/imm.13162. 31792952 PMC7218408

[26] Li Y, Liu H, Zhang L, Yang Y, Lin Y, Zhuo Y, Fang Z, Che L, Feng B, Xu S, Li J, Wu D. 2019. Maternal dietary fiber composition during gestation induces changes in offspring antioxidative capacity, inflammatory response, and gut microbiota in a sow model. Int J Mol Sci 21:31. doi:10.3390/ijms21010031. 31861629 PMC6981455

[27] Wang H. 2016. Effects of exogenous catalase on tissue antioxidant capacity and intestinal microflora. PhD thesis. Jiangnan University, Wuxi, China.

[28] Jones BA, Gores GJ. 1997. Physiology and pathophysiology of apoptosis in epithelial cells of the liver, pancreas, and intestine. Am J Physiol 273:G1174–1188. doi:10.1152/ajpgi.1997.273.6.G1174. 9435542

[29] D’Arcy MS. 2019. Cell death: a review of the major forms of apoptosis. Cell Biol Int 43:582–592. doi:10.1002/cbin.11137. 30958602

[30] Van Opdenbosch N, Lamkanfi M. 2019. Caspases in cell death, inflammation, and disease. Immunity 50:1352–1364. doi:10.1016/j.immuni.2019.05.020. 31216460 PMC6611727

[31] Abate M, Festa A, Falco M, Lombardi A, Luce A, Grimaldi A, Zappavigna S, Sperlongano P, Irace C, Caraglia M, Misso G. 2020. Mitochondria as playmakers of apoptosis, autophagy and senescence. Semin Cell Dev Biol 98:139–153. doi:10.1016/j.semcdb.2019.05.022. 31154010

[32] Alikhani M, Alikhani Z, He H, Liu R, Popek BI, Graves DT. 2003. Lipopolysaccharides indirectly stimulate apoptosis and global induction of apoptotic genes in fibroblasts. J Biol Chem 278:52901–52908. doi:10.1074/jbc.M307638200. 14551216

[33] Tokuda S, Higashi T, Furuse M. 2014. ZO-1 knockout by TALEN-mediated gene targeting in MDCK cells, involvement of ZO-1 in the regulation of cytoskeleton and cell shape. PLoS One 9:e104994. doi:10.1371/journal.pone.0104994. 25157572 PMC4144852

[34] Loboda A, Damulewicz M, Pyza E, Jozkowicz A, Dulak J. 2016. Role of Nrf2/HO-1 system in development, oxidative stress response and diseases, an evolutionarily conserved mechanism. Cell Mol Life Sci 73:3221–3247. doi:10.1007/s00018-016-2223-0. 27100828 PMC4967105

[35] Sajadimajd S, Khazaei M. 2018. Oxidative stress and cancer: The role of Nrf2. Curr Cancer Drug Targets 18:538–557. doi:10.2174/1568009617666171002144228. 28969555

[36] Jiang J, Qi L, Lv Z, Jin S, Wei X, Shi F. 2019. Dietary stevioside supplementation alleviates lipopolysaccharide-induced intestinal mucosal damage through anti-inflammatory and antioxidant effects in broiler chickens. Antioxidants 8:575. doi:10.3390/antiox8120575. 31766443 PMC6943682

[37] Mihara M, Hashizume M, Yoshida H, Suzuki M, Shiina M. 2012. IL-6/IL-6 receptor system and its role in physiological and pathological conditions. Clin Sci (Lond) 122:143–159. doi:10.1042/CS20110340. 22029668

[38] Webb SJ, McPherson JR, Pahan K, Koka S. 2002. Regulation of TNF-alpha-induced IL-6 production in MG-63 human osteoblast-like cells. J Dent Res 81:17–22. doi:10.1177/002203450208100105. 11820362

[39] Goonatilleke E, Smilowitz JT, Mariño KV, German BJ, Lebrilla CB, Barboza M. 2019. Immunoglobulin a n-glycosylation presents important body fluid-specific variations in lactating mothers. Mol Cell Proteomics 18:2165–2177. doi:10.1074/mcp.RA119.001648. 31409668 PMC6823845

[40] Li C, Zhou HC, Nie YL, Zhao BY, Wu CC. 2019. Effects of lipopolysaccharide on T lymphocyte cell subsets and cytokine secretion in mesenteric lymph nodes of mice: Histological and molecular study. Environ Toxicol Pharmacol 71:103214. doi:10.1016/j.etap.2019.103214. 31252312

[41] Perez-Lopez A, Behnsen J, Nuccio SP, Raffatellu M. 2016. Mucosal immunity to pathogenic intestinal bacteria. Nat Rev Immunol 16:135–148. doi:10.1038/nri.2015.17. 26898110

[42] Desai MS, Seekatz AM, Koropatkin NM, Kamada N, Hickey CA, Wolter M, Pudlo NA, Kitamoto S, Terrapon N, Muller A, Young VB, Henrissat B, Wilmes P, Stappenbeck TS, Núñez G, Martens EC. 2016. A dietary fiber-deprived gut microbiota degrades the colonic mucus barrier and enhances pathogen susceptibility. Cell 167:1339–1353e1321. doi:10.1016/j.cell.2016.10.043. 27863247 PMC5131798

[43] Xu X, Hua H, Wang L, He P, Zhang L, Qin Q, Yu C, Wang X, Zhang G, Liu Y. 2020. Holly polyphenols alleviate intestinal inflammation and alter microbiota composition in lipopolysaccharide-challenged pigs. Br J Nutr 123:881–891. doi:10.1017/S0007114520000082. 31928547

[44] Carbonero F, Mayta A, Bolea M, Yu JZ, Lindeblad M, Lyubimov A, Neri F, Szilagyi E, Smith B, Halliday L, Bartholomew A. 2019. Specific members of the gut microbiota are reliable biomarkers of irradiation intensity and lethality in large animal models of human health. Radiat Res 191:107–121. doi:10.1667/RR14975.1. 30430918

[45] Tsatsaronis JA, Walker MJ, Sanderson-Smith ML. 2014. Host responses to group a streptococcus, cell death and inflammation. PLoS Pathog 10:e1004266. doi:10.1371/journal.ppat.1004266. 25165887 PMC4148426

[46] Chen L, Wang W, Zhou R, Ng SC, Li J, Huang M, Zhou F, Wang X, Shen B, M AK, Wu K, Xia B. 2014. Characteristics of fecal and mucosa-associated microbiota in Chinese patients with inflammatory bowel disease. Medicine (Baltimore, MD) 93:e51. doi:10.1097/MD.0000000000000051. PMC4602441 25121355

[47] Murphy EA, Velazquez KT, Herbert KM. 2015. Influence of high-fat diet on gut microbiota, a driving force for chronic disease risk. Curr Opin Clin Nutr Metab Care 18:515–520. doi:10.1097/MCO.0000000000000209. 26154278 PMC4578152

[48] Chaima D. 2020. Characterization of the fecal microbiota of rural Malawian children, associations with biomarkers of environmental enteric dysfunction and the impact of a mass drug administration program. PhD thesis. London School of Hygiene & Tropical Medicine, London, United Kingdom.

[49] Guo M, Wang H, Xu S, Zhuang Y, An J, Su C, Xia Y, Chen J, Xu ZZ, Liu Q, Wang J, Dan Z, Chen K, Luan X, Liu Z, Liu K, Zhang F, Xia Y, Liu X. 2020. Alteration in gut microbiota is associated with dysregulation of cytokines and glucocorticoid therapy in systemic lupus erythematosus. Gut Microbes 11:1758–1773. doi:10.1080/19490976.2020.1768644. 32507008 PMC7524333

[50] Edgar RC. 2013. UPARSE, highly accurate OTU sequences from microbial amplicon reads. Nat Methods 10:996–998. doi:10.1038/nmeth.2604. 23955772

[51] Quast C, Pruesse E, Yilmaz P, Gerken J, Schweer T, Yarza P, Peplies J, Glöckner FO. 2013. The SILVA ribosomal RNA gene database project: improved data processing and web-based tools. Nucleic Acids Res 41:D590–6. doi:10.1093/nar/gks1219. 23193283 PMC3531112

[52] Lawley B, Tannock GW. 2017. Analysis of 16S rRNA gene amplicon sequences using the QIIME software package. Methods Mol Biol 1537:153–163. doi:10.1007/978-1-4939-6685-1_9. 27924593

[53] Lozupone C, Knight R. 2005. UniFrac, a new phylogenetic method for comparing microbial communities. Appl Environ Microbiol 71:8228–8235. doi:10.1128/AEM.71.12.8228-8235.2005. 16332807 PMC1317376

