# Supplementary material for: Supplementation with Exogenous Catalase from Penicillium notatum in the Diet Ameliorates Lipopolysaccharide-Induced Intestinal Oxidative Damage through Affecting Intestinal Antioxidant Capacity and Microbiota in Weaned Pigs
Source: Microbiol Spectr. 2021 Dec 15;9(3):e00654-21. doi: 10.1128/Spectrum.00654-21 (PMC8672903; doi:10.1128/Spectrum.00654-21)
Supplement: SUPPLEMENTAL FILE 1 — Supplemental material. Download SPECTRUM00654-21_Supp_1_seq14.pdf, PDF file, 0.3 MB [file spectrum00654-21_supp_1_seq14.pdf]

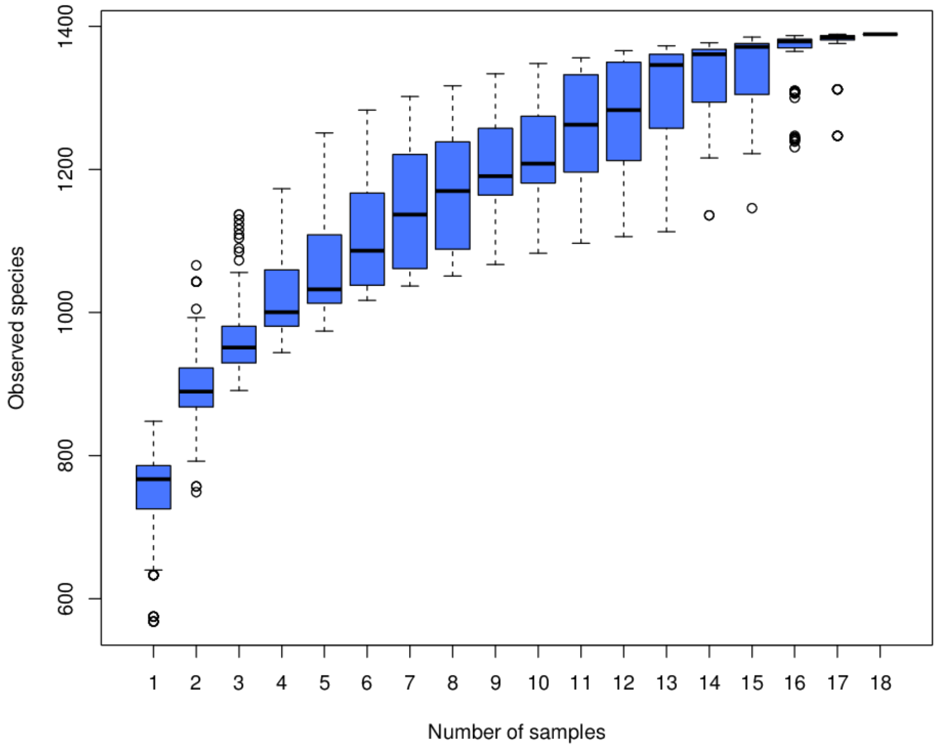


**FIGURE S1.** Species accumulation curves (SAC). The SAC tends to flatten as the number of analyzed sequences increases up to 18, indicating that our samples were sufficient for OTU testing and could predict the species richness of samples.

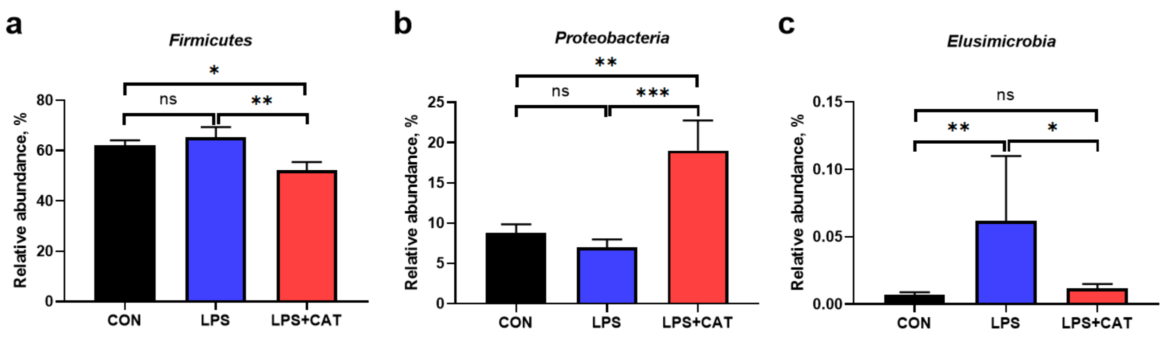


**FIGURE S2.** Effects of dietary supplementation with exogenous catalase (CAT) on the relative abundance of colonic microbiota in phyla in weaned pigs challenged with lipopolysaccharide (LPS). (**a**) *Firmicutes*; (**b**) *Proteobacteria*; (**c**) *Elusimicrobia*. CON, pigs fed the basal diet and given intraperitoneal administration of saline solution; LPS, pigs fed the basal diet and given intraperitoneal administration of LPS; LPS+CAT, pigs fed the basal diet supplemented with 2,000 mg/kg exogenous CAT and given intraperitoneal administration of LPS. The statistical analysis was performed using one-way analysis of variance, and the differences among group means were compared using the least significant difference method. Values are mean ± standard error (N = 6). Significant differences are displayed in the figures by * $p < 0.05$, ** $p < 0.01$ and *** $p < 0.001$, while $0.05 < p < 0.10$ was considered as a trend to significance. Non-significant differences are indicated by “ns”.

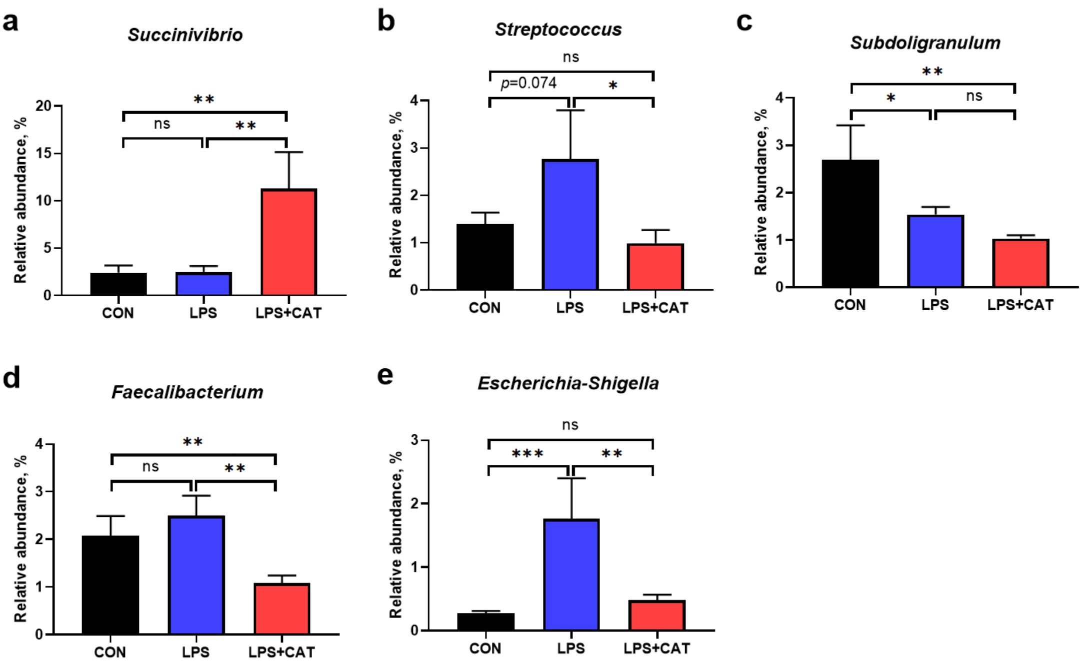


**FIGURE S3**. Effects of dietary supplementation with exogenous catalase (CAT) on the relative abundance of colonic microbiota in genus in weaned pigs challenged with lipopolysaccharide (LPS). (**a**) *Succinivibrio*; (**b**) *Sreptococcus*; (**c**) *Subdoligranulum*; (**d**) *Faecalibacterium*; (**e**) *Escherichia-Shigella*. CON, pigs fed the basal diet and given intraperitoneal administration of saline solution; LPS, pigs fed the basal diet and given intraperitoneal administration of LPS; LPS+CAT, pigs fed the basal diet supplemented with 2,000 mg/kg exogenous CAT and given intraperitoneal administration of LPS. The statistical analysis was performed using one-way analysis of variance, and the differences among group means were compared using the least significant difference method. Values are mean ± standard error (N = 6). Significant differences are displayed in the figures by * $p < 0.05$, ** $p < 0.01$, and *** $p < 0.001$, while $0.05 < p < 0.10$ was considered as a trend to significance. Non-significant differences are indicated by “ns”.

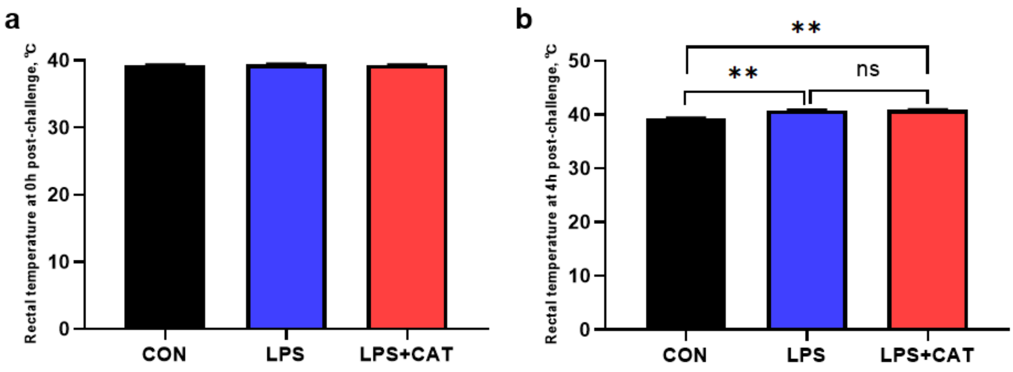


**FIGURE S4.** Rectal temperature of weaned pigs after lipopolysaccharide (LPS) or sterile saline injection. CON, pigs fed the basal diet and given intraperitoneal administration of saline solution; LPS, pigs fed the basal diet and given intraperitoneal administration of LPS; LPS+CAT, pigs fed the basal diet supplemented with 2,000 mg/kg exogenous CAT and given intraperitoneal administration of LPS. (**a**) Rectal temperature 0 h after LPS or sterile saline injection; (**b**) Rectal temperature 4 h after LPS or sterile saline injection. The statistical analysis was performed using one-way analysis of variance, and the differences among group means were compared using the least significant difference method. Values are mean ± standard error (N = 6). Significant differences are displayed in the figures by * $p < 0.05$ and ** $p < 0.01$. Non-significant differences are indicated by "ns". No significant difference was observed in rectal temperature among the three treatments 0 h after LPS or sterile saline injection ($p > 0.05$), but LPS administration resulted in an increase in rectal temperature at 4 h post-challenge compared with the CON piglets ($p < 0.05$), indicating that LPS challenge model was established successfully.

**TABLE S1.** Operational taxonomic unit (OTU) clustering and annotation per sample.

| Samples[1] | Total tags | Taxon tags | Unclassified tags | Unique tags | OUT numbers |
|---|---|---|---|---|---|
| CON1 | 76530 | 74356 | 0 | 2174 | 870 |
| CON2 | 81684 | 80123 | 3 | 1558 | 628 |
| CON3 | 78021 | 75813 | 0 | 2208 | 846 |
| CON4 | 62491 | 61008 | 1 | 1482 | 806 |
| CON5 | 79907 | 77998 | 0 | 1909 | 687 |
| CON6 | 76417 | 74055 | 0 | 2362 | 885 |
| LPS1 | 65620 | 64224 | 0 | 1396 | 830 |
| LPS2 | 76103 | 73497 | 0 | 2606 | 906 |
| LPS3 | 68435 | 66440 | 0 | 1995 | 837 |
| LPS4 | 72316 | 71249 | 0 | 1067 | 623 |
| LPS5 | 67185 | 65743 | 0 | 1442 | 819 |
| LPS6 | 79738 | 77648 | 0 | 2090 | 845 |
| LPS+CAT1 | 67153 | 65986 | 0 | 1167 | 767 |
| LPS+CAT2 | 64036 | 62113 | 0 | 1923 | 817 |
| LPS+CAT3 | 54911 | 53798 | 1 | 1112 | 641 |
| LPS+CAT4 | 67727 | 65690 | 0 | 2037 | 847 |
| LPS+CAT5 | 66957 | 65082 | 0 | 1875 | 823 |
| LPS+CAT6 | 63537 | 61805 | 0 | 1732 | 826 |

[1]CON 1, 2, 3, 4, 5, and 6 means colonic digesta samples from pigs fed with basal diet

and given intraperitoneal administration of saline solution; LPS 1, 2, 3, 4, 5, and 6 means colonic digesta samples from pigs fed the basal diet and given intraperitoneal administration of lipopolysaccharide; LPS+CAT1, 2, 3, 4, 5, and 6 means colonic digesta samples from piglets fed the basal diet supplemented with 2.0 g/kg exogenous CAT and given intraperitoneal administration of lipopolysaccharide.

**TABLE S2**. Ingredients composition and nutrient levels of basal diets (as-fed basis).

| Items | Phases | |
|---|---|---|
| | **1-21 d** | **22-35 d** |
| Ingredients, % | | |
| Corn | 37.55 | 48.09 |
| Extruded corn | 18.00 | 15.00 |
| Soybean meal | 13.00 | 18.50 |
| Extruded soybean | 10.00 | 6.00 |
| Fish meal | 4.00 | 3.00 |
| Spray-dried plasma protein | 3.00 | 0.00 |
| Whey powder | 10.00 | 5.00 |
| Soy oil | 1.03 | 1.08 |
| Monocalcium phosphate | 0.78 | 0.66 |
| Limestone | 0.95 | 0.90 |
| NaCl | 0.30 | 0.30 |
| L-Lysine HCl | 0.32 | 0.39 |
| DL-Methionine | 0.16 | 0.20 |
| L-Threonine | 0.11 | 0.16 |
| L-Tryptophan | 0.00 | 0.02 |
| Corn starch | 0.30 | 0.20 |
| Vitamin-mineral premix [1] | 0.50 | 0.00 |
| Vitamin-mineral premix [2] | 0.00 | 0.50 |

| | | |
|---|---|---|
| Total | 100.00 | 100.00 |
| Nutrient composition [3] | | |
| Digestible energy, Mcal/kg | 3.54 | 3.49 |
| Crude protein, % | 20.56 | 18.88 |
| Ca, % | 0.80 | 0.70 |
| Digestible P, % | 0.40 | 0.34 |
| Lysine, % | 1.35 | 1.24 |
| Methionine, % | 0.39 | 0.36 |
| Threonine, % | 0.79 | 0.73 |
| Tryptophan, % | 0.23 | 0.20 |

[1] The premix provided for per kg of feed: Zn, 100 mg; Mn, 4 mg; Fe, 100 mg; Cu, 6 mg; I, 0.14 mg; Se, 0.3 mg; choline chloride, 500 mg; vitamin A, 10,500 IU; vitamin D3, 3,300 IU; vitamin E, 22.5 IU; vitamin K3, 3 mg; vitamin B1, 3 mg; vitamin B2, 7.5 mg; vitamin B6, 4.5 mg; vitamin B12, 0.03 mg; niacin, 30 mg; pantothenate, 15 mg; folic acid, 1.5 mg; biotin, 0.12 mg.

[2] The premix provided for per kg of feed: Zn, 80 mg; Mn, 3 mg; Fe,100 mg; Cu, 5 mg; I, 0.14 mg; Se, 0.25 mg; choline chloride, 400 mg; vitamin A, 10,500 IU; vitamin D3, 3,300 IU; vitamin E, 22.5 IU; vitamin K3, 3 mg; vitamin B1, 3 mg; vitamin B2, 7.5 mg; vitamin B6, 4.5 mg; vitamin B12, 0.03 mg; niacin, 30 mg; pantothenate, 15 mg; folic acid, 1.5 mg; biotin, 0.12 mg.

[3] All data were calculated according to the tables of Feed Composition and Nutrient Values in China (2016) in two diets.

**TABLE S3.** Primer sequence used for quantitative real-time PCR.

| Genes | Primer sequences [1] (5′-3′) | Product Size, bp | Gene bank No. |
|---|---|---|---|
| *GAPDH* | F: TCGGAGTGAACGGATTTGGC<br>R: TGCCGTGGGTGGAATCATAC | 147 | NM_001206359.1 |
| Nrf2 | F: GCCCCTGGAAGCGTTAAAC<br>R: GGACTGTATCCCCAGAAGGTTGT | 67 | XM_021075133.1 |
| HO-1 | F: AGCTGTTTCTGAGCCTCCAA<br>R: CAAGACGGAAACACGAGACA | 130 | NM_001004027.1 |
| NF-κB | F: AGTACCCTGAGGCTATAACTCGC<br>R: TCCGCAATGGAGGAGAAGTC | 133 | NM_001114281.1 |
| *ZO-1* | F: CAGAGACCAAGAGCCGTCC<br>R: TGCTTCAAGACATGGTTGGC | 105 | XM-003480423.4 |
| *Occludin* | F: TCAGGTGCACCCTCCAGATT<br>R: AGGAGGTGGACTTTCAAGAGG | 118 | NM-001163647.2 |
| *Claudin-1* | F: ATTTCAGGTCTGGCTATCTTAGTTGC<br>R: AGGGCCTTGGTGTTGGGTAA | 214 | NM-001244539.1 |

[1] F, forward; R, reverse.
